# Phylogenetic analyses reveal molecular signatures associated with functional divergence among Subtilisin like Serine Proteases are linked to lifestyle transitions in Hypocreales

**DOI:** 10.1186/s12862-016-0793-y

**Published:** 2016-10-19

**Authors:** Deepti Varshney, Akanksha Jaiswar, Alok Adholeya, Pushplata Prasad

**Affiliations:** TERI Deakin Nanobiotechnology Centre, TERI Gram, The Energy and Resources Institute, Gual Pahari, Faridabad Road, Gurgaon, Haryana 122001 India

**Keywords:** *P. lilacinum*, Subtilisin like serine proteases, Phylogenetic analysis, Gene tree, Species tree, Conserved motif analysis, Natural selection, Type I functional divergence, Type II functional divergence, Protein structure modeling

## Abstract

**Background:**

Subtilisin-like serine proteases or Subtilases in fungi are important for penetration and colonization of host. In Hypocreales, these proteins share several properties with other fungal, bacterial, plant and mammalian homologs. However, adoption of specific roles in entomopathogenesis may be governed by attainment of unique biochemical and structural features during the evolutionary course. Due to such functional shifts Subtilases coded by different family members of Hypocreales acquire distinct features according to respective hosts and lifestyle. We conducted phylogenetic and DIVERGE analyses and identified important protein residues that putatively assign functional specificity to Subtilases in fungal families/species under the order Hypocreales.

**Results:**

A total of 161 Subtilases coded by 10 species from five different families under the fungal order Hypocreales was included in the analysis. Based on the presence of conserved domains, the Subtilase genes were divided into three subfamilies, Subtilisin (S08.005), Proteinase K (S08.054) and Serine-carboxyl peptidases (S53.001). These subfamilies were investigated for phylogenetic associations, protein residues under positive selection and functional divergence among paralogous clades. The observations were co-related with the life-styles of the fungal families/species. Phylogenetic and Divergence analyses of Subtilisin (S08.005) and Proteinase K (S08.054) families of proteins revealed that the paralogous clades were clear-cut representation of familial origin of the protein sequences. We observed divergence between the paralogous clades of plant-pathogenic fungi (Nectriaceae), insect-pathogenic fungi (Cordycipitaceae/Clavicipitaceae) and nematophagous fungi (Ophiocordycipitaceae). In addition, Subtilase genes from the nematode-parasitic fungus *Purpureocillium lilacinum* made a unique cluster which putatively indicated that the fungus might have developed distinctive mechanisms for nematode-pathogenesis. Our evolutionary genetics analysis revealed evidence of positive selection on the Subtilisin (S08.005) and Proteinase K (S08.054) protein sequences of the entomopathogenic and nematophagous species belonging to Cordycipitaceae, Clavicipitaceae and Ophiocordycipitaceae families of Hypocreales.

**Conclusions:**

Our study provided new insights into the evolution of Subtilisin like serine proteases in Hypocreales, a fungal order largely consisting of biological control species. Subtilisin (S08.005) and Proteinase K (S08.054) proteins seemed to play important roles during life style modifications among different families and species of Hypocreales. Protein residues found significant in functional divergence analysis in the present study may provide support for protein engineering in future.

**Electronic supplementary material:**

The online version of this article (doi:10.1186/s12862-016-0793-y) contains supplementary material, which is available to authorized users.

## Background

The fungal order Hypocreales includes a wide range of ecologically diverse species including plant-pathogens, plant-endophytes, mycoparasites, and pathogens of insects and nematodes [1]. Several Hypocrealean species possesses effective pathogenic mechanisms and have been commercialized as bio-pesticides for plant-pathogens [2, 3]. Hypocrealean fungi are reported to display substantial flexibility of lifestyles [4]. Large phylogenetic studies suggest that multiple transitions between different lifestyles have remained events of considerable importance in the evolutionary history of these fungi [5, 6]. These fungi adapt to the changed environmental conditions when switching hosts and habitats putatively by acquiring proteolytic genes, such as Subtilases [4]. Subtilases are characterized by the presence of catalytic triad (Asp-His-Ser) and utilize a catalytic Ser residue for activity [7]. Evolutionarily, Subtilases are conserved in all three domains of life, Archaea, Bacteria and Eukaryotes [8, 9]. Subtilases in Hypocrealean fungi play integral roles in host pathogenesis such as degradation of insect cuticle or protein-containing component of the egg shell [10, 11]. Although, Subtilases are largely conserved in different life forms, they exhibit genetic and functional dissimilarities between different fungal genomes [12].

Recent whole genome sequencing efforts have identified Subtilase genes present in various Hypocrealean species [13–16]. Further, gene expression studies have emphasized on putative involvement of these Subtilases in pathogenicity. Subtilisin-like serine proteases produced by *Purpureocillium lilacinum* are reported to degrade protein components of nematode and insect eggs. These proteins also play important role in the evolution of pathogenicity of nematode-trapping fungi against nematodes [12]. In a previous study, we reported that 61 serine protease genes in the *P. lilacinum* genome had homologs present in the pathogen – host interaction (PHI) database, which supported their role in pathogenicity [4]. In nematophagous fungus *Pochonia chlamydosporia*, 59 % of serine proteases showed expression during their endophytic interaction with host [16]. Enzymes involved in an organism’s response to pathogens and environmental stresses are among the functional categories most prone to expansion [17]. Subtilisins are found expanded in *Metarhizium* with lineage-specific duplications and had significant matches in the PHI-database [14]. Similarly, Subtilisin proteins mediated the infection processes by degrading host cuticles in *Metarhizium anisopliae* [18]. Subtilisins involved in degrading insect cuticles are found expanded in *Beauveria bassiana* and *Cordyceps militaris* [19, 20].

Despite previous efforts invested in identifying the important functional variations between different Subtilases expressed by Hypocreales, underlying molecular mechanisms involved in life style adaptation and pathogenesis remain elusive. This requires exhaustive exploration to identify protein residues under natural selection pressure in these genes. To date, only a few limited studies on the evolutionary pattern of Subtilases in entomopathogenic fungi [21–23] have been carried out. No detailed bioinformatics analyses have been performed to correlate the evolutionary dynamic differences between Subtilases coded by different families under Hypocreales and functional shift.

In this study we conducted advanced bioinformatics analyses to elucidate the critical selective constraints leading to functional differentiation between Subtilases of different families belonging to Hypocreales. In accordance with current classification, Subtilases were divided into two families: Protease S08 family of the Subtilase-like protease and S53 family of Serine-carboxyl peptidases. The family S08 was further grouped into two subfamilies, Subtilisin (S08.005) and Proteinase K (S8.054). To identify functional divergence between paralogous proteins present in separate phylogenetic clusters, we evaluated selective constraints (residues under positive selection) after gene duplication, and mapped amino acid sites involved in functional divergence on secondary and tertiary structures of selected Subtilases. The effects of amino acid sites involved in functional divergence on functional shift and structural stability of the proteins were discussed.

Findings of the present study could provide important insights into the evolution of pathogenic mechanisms in different families of Hypocreales. Observations made in the study could be further translated for engineering Subtilases with customized biotechnological properties for application in biological control and waste treatment.

## Results and Discussion

### Phylogenetic analysis

The order Hypocreales consist of seven families: Nectriaceae, Cordycipitaceae, Clavicipitaceae, Ophiocordycipitaceae, Hypocreaceae, Bionectriaceae and Niessliaceae. Whole Genome sequencing (WGS) and genome annotation have been done for multiple species belonging to the five families i.e., Nectriaceae, Cordycipitaceae, Clavicipitaceae, Ophiocordycipitaceae and Hypocreaceae [4]. However, very limited information is present in the NCBI database for the remaining two families with only one species sequenced for each, Bionectriaceae: *Clonostachys rosea* and Niessliaceae: *Niessliaceae Valetoniellopsis laxa*. Therefore, we carried out phylogenetic analyses by using the whole genome sequence and annotation files (available in NCBI db) of 10 representative fungal species belonging only to the five families, Nectriaceae, Cordycipitaceae, Clavicipitaceae, Ophiocordycipitaceae and Hypocreaceae. Clavicipitaceae, Cordycipitaceae, and Ophiocordycipitaceae families are particularly rich in entomopathogenic species. Families Nectriaceae and Hypocreaceae contain plant pathogenic and mycoparasitic species respectively. The Subtilase proteins coded by different species of Hypocreales were identified by using MEROPS (https://merops.sanger.ac.uk/) [24].

In Nectriaceae family, 27 and 45 Subtilase proteins coded by *Fusarium graminearum* and *Fusarium oxysporum* genomes respectively were identified. In Cordycipitaceae family, 41 and 33 Subtilase proteins belonging to *Beauveria bassiana* and *Cordyceps militaris* genomes respectively were recorded. In Clavicipitaceae family, 42 Subtilases of *Metarhizium acridum*, 57 of *Metarhizium robertsii* and 28 of *Pochonia chlamydosporia* were identified. In Ophiocordycipitaceae family, 23 and 44 Subtilase proteins among *Tolypocladium inflatum* and *Purpureocillium lilacinum* genomes were found. The genome of *Trichoderma reesei* belonging to Hypocreaceae family coded for 22 Subtilases.

**Fig. 1:**
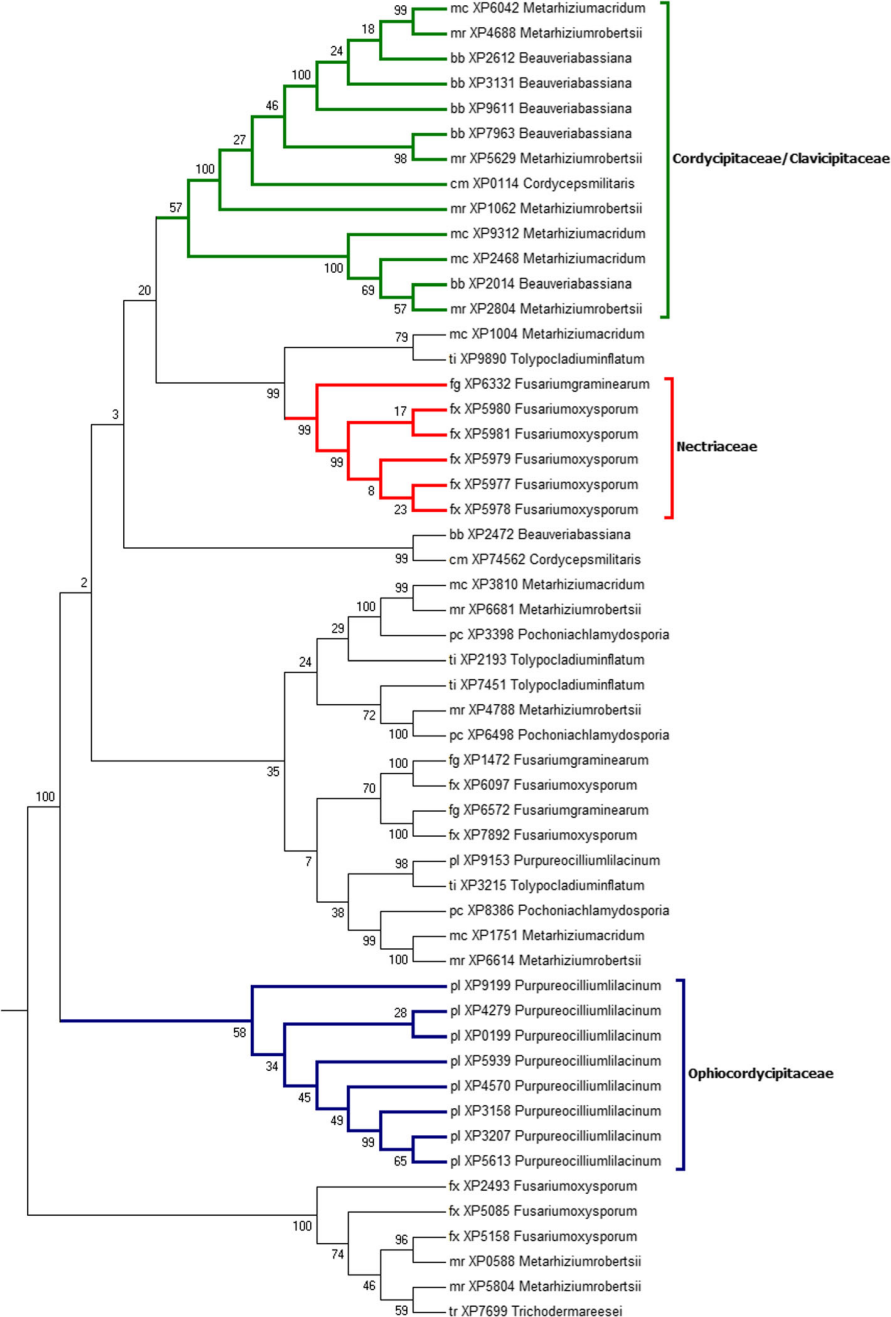
Phylogenetic relationships among protein sequences belonging to the Subtilisin (S08.005) family. The numbers indicate the Bootstrap values for each branch

**Fig. 2:**
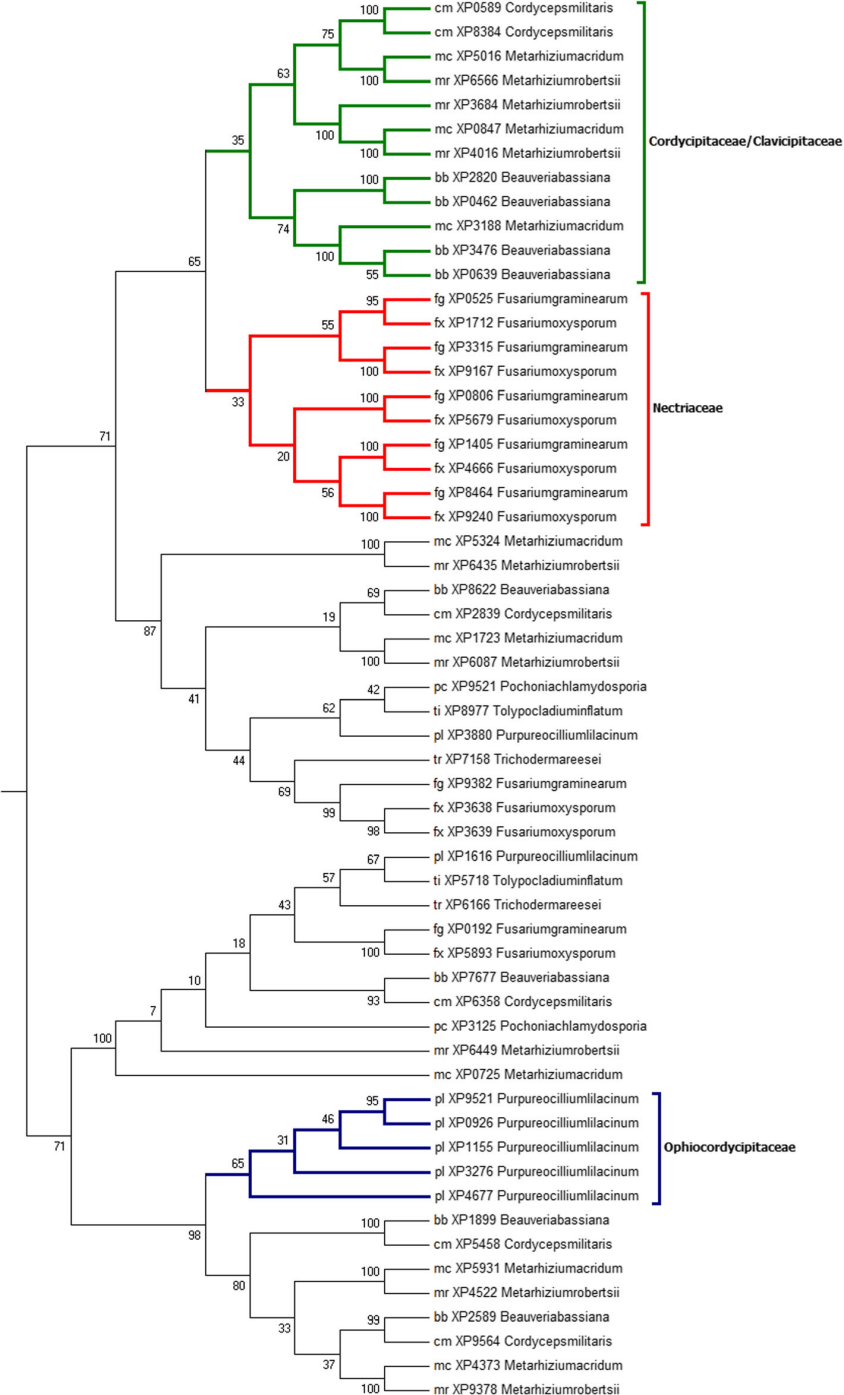
Phylogenetic relationships among protein sequences belonging to the Proteinase K (S08.054) family. The numbers indicate the Bootstrap values for each branch

Based on homology and motif search, these proteins were grouped under three subfamilies, Subtilisins (S08.005, no. of proteins = 53), Proteinase K (S08.054, no. of proteins =58) and Serine-carboxyl peptidases (S53.001, no. of proteins = 50). Additional file 1: Table S1 provides the accession details of these protein sequences. Sequences were subjected to multiple sequence alignments and a maximum likelihood (ML) phylogenetic tree was constructed in MEGA 6.0 (http://www.megasoftware.net/). Stabilized phylogenetic trees for Subtilisin (S08.005), Proteinase K (S08.054) and Serine-carboxyl peptidase (S53.001) proteins are presented in Figs. 1 and 2 and Additional file 2: Figure S1 respectively.

### Phylogenetic analysis of the Subtilisin (S08.005) gene family

The consensus phylogeny obtained for Subtilisin (S08.005) family protein sequences is shown in Fig. 1. The protein sequences from 10 species were clustered into six orthologous clades. Three clades namely, “Ophiocordycipitaceae”, “Nectriaceae” and “Cordycipitaceae/Clavicipitaceae”, consisted of protein sequences from corresponding families of Hypocreales. These three clades categorically reflected familial origin of the proteins. However, the remaining three clades possessed protein members from multiple families of Hypocreales and thus seemed to be conserved across families. The first family based clade “Ophiocordycipitaceae” consisted exclusively of 8 Subtilisin (S08.005) sequences of *P. lilacinum* which is a nematophagous fungus belonging to the family Ophiocordycipitaceae. The second clade “Nectriaceae” consisted of 6 members of *F. graminearum* and *F. oxysporum* that are known plant pathogens. The third Clade consisted of 13 members of Subtilisin (S08.005) sequences of insect pathogens (*B. bassiana, C. militaris, M. acridum, M. robertsii*) belonging to the families Cordycipitaceae and Clavicipitaceae.

Such a distribution of protein sequences in the phylogenetic tree provided insights into the evolutionary history of Subtilisin (S08.005) protein family in Hypocreales. The composition of the three family oriented clades in the phylogenetic tree suggested that Subtilases in Hypocreales evolved to support lifestyle shifts from plant-pathogenesis (Nectriaceae) to insect-pathogenesis (Cordycipitaceae/Clavicipitaceae) on one hand and to nematophagy (Ophiocordycipitaceae) on the other hand. Another noteworthy observation was a distinct composition of “Ophiocordycipitaceae” clade that was composed of protein sequences exclusively of *P. lilacinum*, which is a nematode trapping fungus. One of the previous studies on nematode trapping fungi concluded that positive selection acted on the Subtilisin-like serine protease genes in nematode-trapping fungi, at least in the early stage of their evolution, which probably helped them diverge and acquire life-style specific functions. Furthermore, separate clustering of the nematode trapping fungi is in agreement with previous reports [12, 21–23]. The composition of the “Ophiocordycipitaceae” clade in the phylogenetic tree constructed in this study argued that in order to attain functional features for trapping nematode, Subtilisin (S08.005) protein sequences in *P. lilacinum* could have acquired positively selected residues and originated independently of sequences in plant pathogenic and entomopathogenic families of Hypocreales. Protein sequences of *T. inflatum,* another member of Ophiocordycipitaceae family, were not clustered in the “Ophiocordycipitaceae” clade and were found distributed across the phylogenetic tree. *T. inflatum* is primarily a pathogen of beetle larvae [25], which also exists as a soil-saprotrophyte during the asexual phase of its lifecycle. Such a phylogenetic distribution of protein sequences of *T. inflatum* could indicate a robust capability of its proteome to adapt to multiple and variable life strategies [1] according to the changed environment. The Subtilisin (S08.005) proteins in these two species of Ophiocordycipitaceae could have diverged quite a long time back and acquired functional differences.

### Phylogenetic analysis of the proteinase K (S08.054) gene family

The Proteinase K family was first identified in the fungi *Tritirachium album* and named for its similarity to the widely known *T. album* proteinase K [26]. These proteases are generally characterized by the presence of a Subtilisin N-terminal domain containing a propeptide (which is thought to act as an intra-molecular chaperone to assist protein folding as well as inhibit enzyme activity) and a catalytic peptidase S8 domain.

The paralogous clades in phylogenetic tree seemed to differ from each other mainly by the presence of members belonging to specific families. Similar to Subtilisin (S08.005) protein family, the phylogenetic tree clusters the selected 58 Proteinase K (S08.054) protein sequences into three family based orthologous clades: “Ophiocordycipitaceae”, “Nectriaceae” and “Cordycipitaceae/Clavicipitaceae” (Fig. 2). Also similar to Subtilisin (S08.005) (Fig. 1), 5 sequences of Proteinase K (S08.054) of *P. lilacinum* constructed a separate clade “Ophiocordycipitaceae”. This was in agreement with previous studies [12, 21–23], where in subtilases of *P. lilacinum* genome clustered distinctly from another member of the same family (*T. inflatum*) and the other nematophagous fungus (*P. chlamydosporia*) included in our study. A total of 10 Proteinase K (S08.054) sequences, 5 from *F. graminearum* and 5 from *F. oxyspo*rum composed the “Nectriaceae” clade. The “Cordycipitaceae/Clavicipitaceae” clade consisted of a total of 12 proteins sequences from four representative species (*B. bassiana, C. militaris, M. robertsii and M. acridum)* of Cordycipitaceae and Clavicipitaceae families included in the study.

Placement of clades with respect to each other seemed intriguing. In the phylogenetic tree “Nectriaceae” clade of plant pathogens was arranged between the “Ophiocordycipitaceae” and “Cordycipitaceae/Clavicipitaceae” clades of nematophagous fungus and insect pathogens respectively. This kind of arrangement of the paralogous clades of Proteinase K (S08.054) sequences (dissimilar to the species tree [4]) putatively indicated towards divergent evolution of Proteinase K (S08.054) sequences to provide fitness to the fungal species according to the changes in host and habitat.

### Phylogenetic analysis of the Serine-carboxyl peptidases (S53.001) family

Serine-carboxyl peptidases (S53.001) family is also called as Sedolisins. The protein folds of S53.001 peptidases resembles that of Subtilisin (S08.005), however they are considerably larger, with the mature catalytic domains containing approximately 375 amino acids. These proteins possess unique catalytic triad, Ser-Glu-Asp as well as the presence of an aspartic acid residue in the oxyanion hole.

In the present study, no distinct family based clades were observed in the phylogenetic analysis of the Serine-carboxyl peptidases (S53.001) sequences coded by the 10 fungal species (Additional file 2: Figure S1). Such phylogenetic arrangement of protein sequences may imply insignificant contribution of Serine-carboxyl peptidases (S53.001) towards functional diversification of species in the Hypocreales order. Sequences belonging to the Serine-carboxyl peptidase (S53.001) family were not analyzed further in this study.

### Estimating gene gain and loss via gene tree/species tree Reconciliation

An important consideration in phylogenetic analysis is to address origin of new genes and function among species. Evolutionary history exerts a strong influence on gene function [27, 28] and therefore, accurate inference of gene history is essential. Furthermore, duplication and loss events lead to discordance between the topologies of gene tree and species tree [29]. To address differences between topologies of species tree and gene trees we inferred the history of gene gain and loss among genomes using a parsimony method (NOTUNG) [30] which reconciled the gene tree with the species tree. For this analysis, we used the same species tree that was constructed for the comparative genome analysis of 10 Hypocreales genomes in our previous study [4].

### Gene gain and losses in Subtilisin (S08.005) gene family

**Fig. 3:**
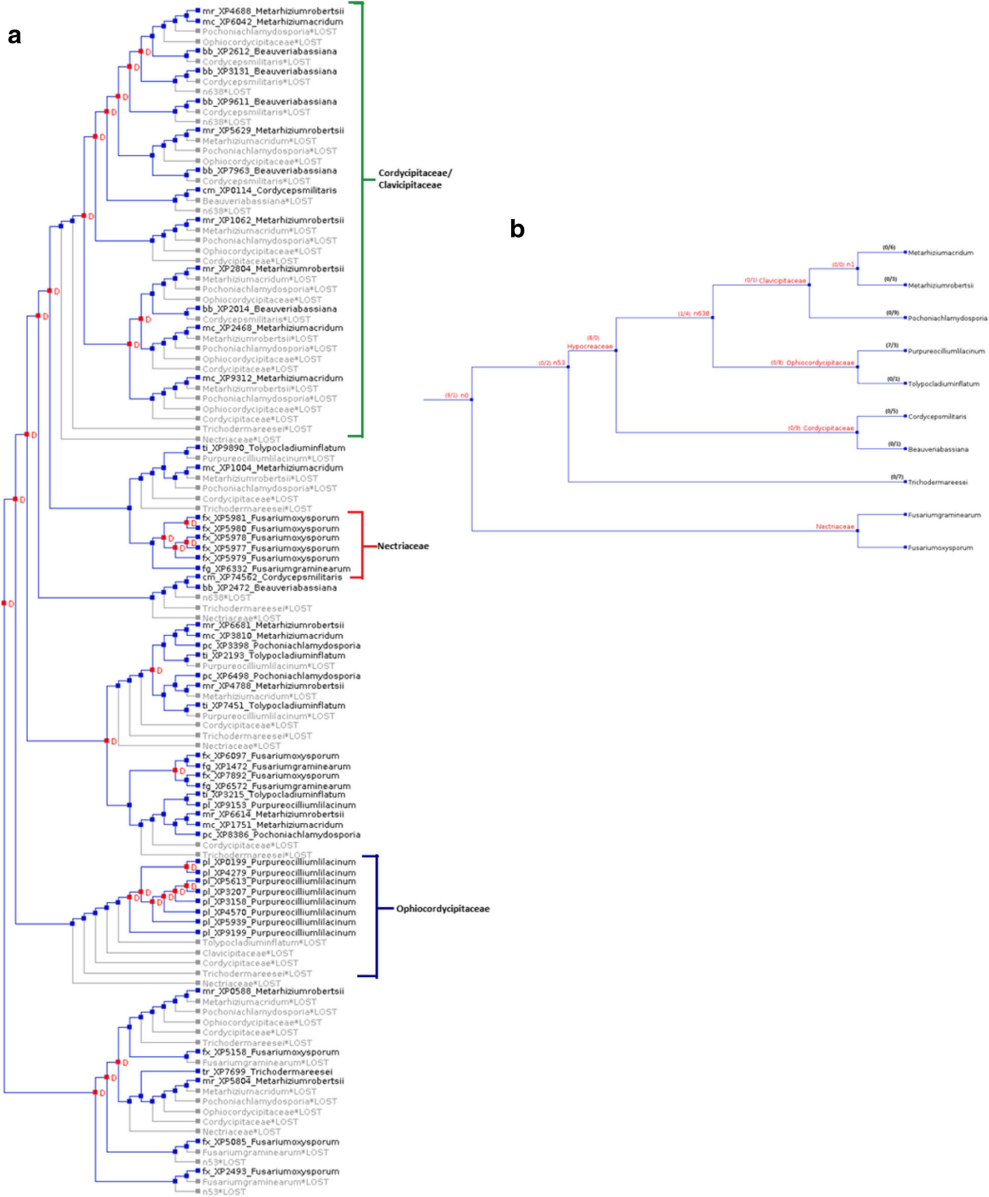
Reconciliation of Subtilisin (S08.005) gene tree with species tree by NOTUNG. **a** Gene tree showing duplications (red boxes and D's) and losses (grey), (**b**) Distribution of Subtilisin (S08.005) gene gains/losses among 10 Hypocreales species

To estimate gene gains and losses in Subtilisin (S08.005) gene family we reconciled the Subtilisin (S08.005) gene tree with the species tree. A total of 30 and 67 events of gene gains and losses respectively were identified. Mapping of gene gains and losses on the three paralogous clades constructed in the phylogenetic tree (Fig. 3) revealed 8 genes gains and 31 gene losses in “Cordycipitaceae/Clavicipitaceae”, 4 gene gains in “Nectriaceae”, 7 gene gains and 5 gene losses in “Ophiocordycipitaceae” clades. Furthermore, gene tree was mapped on the species tree and species wise gene gains and losses were predicted (Fig. 3). NOTUNG also predicted a few lost genes in unrecognizable species (n1, n58, n632) that depicted gene gains and losses in an ancestral species. The ‘rearrange mode’ in NOTUNG, which minimizes the weighted sum of gene gains and losses based upon the threshold value (90), predicted a total of 23 and 33 gene duplication and loss events in the Subtilisin (S08.005) gene tree (Additional file 3: Figure S2).

### Gene gains and losses in Proteinase K (S08.054) gene family

**Fig. 4:**
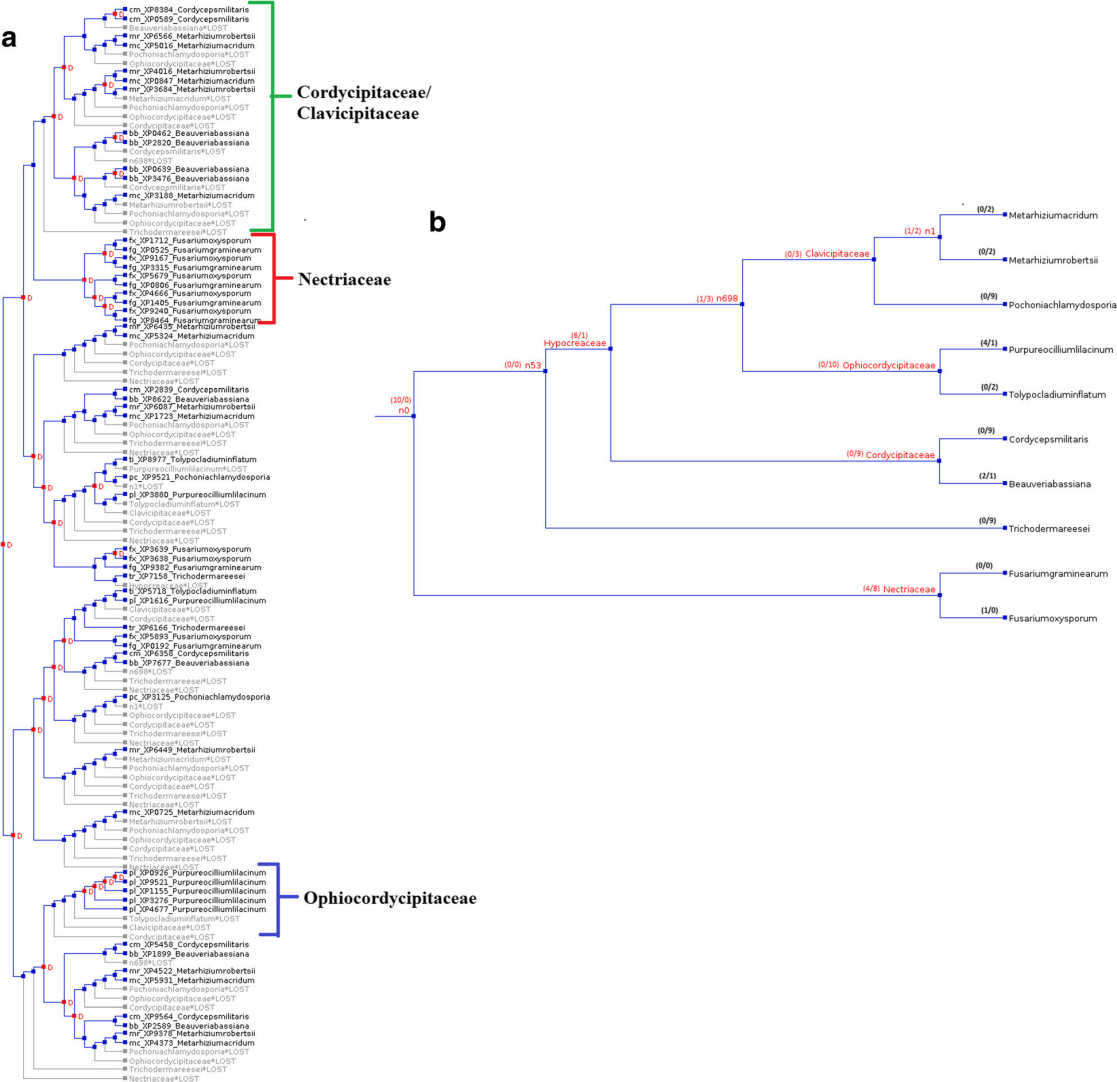
Reconciliation of Proteinase K (S08.054) gene tree with species tree by NOTUNG. **a** Gene tree showing duplications (red boxes and D's) and losses (grey), (**b**) Distribution of Proteinase K (S08.054) gene gains/losses among 10 Hypocreales species

In order to find out gene gains and gene losses in Proteinase K (S08.054) gene family, the gene tree was reconciled with the species tree [4]. A total of 30 and 64 gene gains loss events respectively were identified. Among the three paralogous clades of Proteinase K (S08.054), 14 gene losses and 7 gene gains in “Cordycipitaceae/Clavicipitaceae”, 4 gene gains in “Nectriaceae”, 4 gene gains and 3 gene losses in “Ophiocordycipitaceae” clades respectively were identified (Fig. 4).

Proteinase K (S08.054) gene tree was mapped on the species tree and species wise gene gains and losses were predicted (Fig. 4). Gene losses that occurred in evolutionary history of the species under analysis and also in unrecognizable but ancestral species (n0, n1, n58, n698) were also estimated. In ‘rearrange mode’ a total of 19 and 11 gene gains and gene losses were predicted in Proteinase K gene (S08.054) tree (Additional file 4: Figure S3).

The phylogenetic tree analyses along with gene gain and loss estimation by NOTUNG suggested that gene gain and loss events during the course of evolution perhaps resulted in discordance in tree topologies of the gene trees and species tree in the study.

### Analysis of conserved motifs

To identify differences between the functional and conserved motifs of Subtilases proteins among the family members of Hypocreales in depth exploration was carried out by using Multiple EM for Motif Elicitation (MEME) tool (http://meme-suite.org/) [31].

### Conserved motifs and functionally important residues in Subtilisin (S08.005) family

**Fig. 5:**
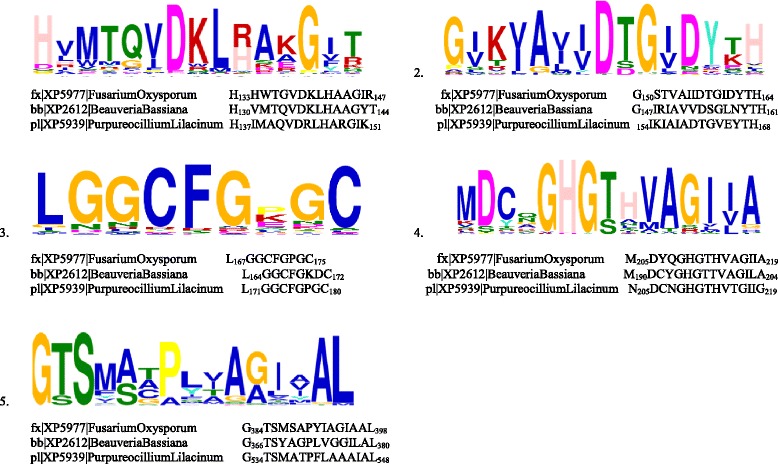
Multiple EM for Motif Elicitation (MEME) analysis for identification of functional motifs among Subtilisin S08.005 protein sequences in Hypocreales

Sequences of all 53 Subtilisin (S08.005) proteins that were included in the phylogenetic analysis were subject to MEME suite. Five distinct motifs were identified to be conserved among all the sequences (Fig. 5 and Additional file 5: Figure S4). Independent inspection of these 5 motifs in 53 sequences seemed to possess variability. However, at the Clade level these 5 motifs were generally conserved in all three paralogous clades of the phylogenetic tree (Fig. 1).

The first two motifs M1 (DKLxxxG) and M2 (AxxDxGxD) were localized in N-terminus of the proteins. The mid region of motif M1 was highly variable among all clades. However, mid region of M2 was conserved within the specific clades. Motif M2 was absent in one (fx|XP5981|*Fusariumoxysporum*) out of the total 53 sequences used in the analysis. In all these clades all “x” positions in motif M2 were occupied by a non-polar residue.

The third motif M3 (LGGCFGxxC) was also present towards the N-terminus of the Subtilisin (S08.005) protein family. The first “x” position was occupied by a negatively charged residue E (Glutamate) in “Ophiocordycipitaceae” clade, a nonpolar residue P (Proline) in the “Cordycipitaceae/Clavicipitaceae” clade and by a positively charged residue K (Lysine) in “Nectriaceae” clade. The second “x” position of this motif was occupied by a non-polar residue G (Glycine) in “Ophiocordycipitaceae” and “Nectriaceae” clades where as in “Cordycipitaceae/Clavicipitaceae” clade it was represented by a negatively charged amino acid residue D (Aspartate).

The fourth motif M4 (GHGxxVAG) was found to be highly conserved in the mid region of Subtilisin (S08.005) proteins. Alanine, a non-polar residue of this motif was replaced by polar residue T/S (Threonine/serine) in “Ophiocordycipitaceae” clade. The first “x” position contained a polar residue T (Threonine) in almost all members of the 3 clades and the second “x” position of this motif was represented by a positively charged residue in the “Ophiocordycipitaceae” and “Nectriaceae” clades whereas in the “Cordycipitaceae/Clavicipitaceae” clade it was occupied by a non-polar residue A/I (Alanine/Isoleucine).

The fifth motif M5 (GTSxxxP), starts with the conserved residues Glycine, Threonine and Serine and is present in mid region of the protein. Although the variable region showed many changes among 53 sequences, it was conserved within the respective paralogous clade.

### Conserved motifs and functionally important residues in Proteinase K (S08.054) family

**Fig. 6:**
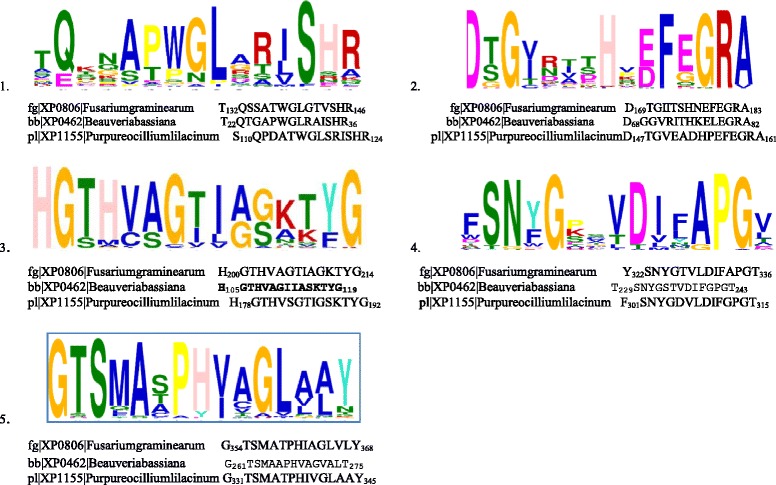
Multiple EM for Motif Elicitation (MEME) analysis for identification of functional motifs among Proteinase K (S08.054) protein sequences in Hypocreales

Protein sequences of 58 proteinase K (S08.054) members were subjected to MEME suite for motif identification. Five distinct motifs were found conserved among all the sequences (Fig. 6 and Additional file 6: Figure S5). Out of these 5 motifs, 2 motifs were the same as the motifs identified in the analysis of Subtilisin (S08.005) protein sequences, namely M3 (HGTxVAG) & M5 (GTSxAxP) at amino acid positions190-220 and 380–550 respectively.

Motif M1 (GLxxxS) was present in the mid region of Proteinase K (S08.054) proteins and was found quite conserved in the three paralogous clades (Nectriaceae, Cordycipitaceae/Clavicipitaceae, and Ophiocordycipitaceae). The variable “xxx” part of M1 seemed to differ in a clade specific manner. In the clade “Ophiocordycipitaceae” the first two “xx” were occupied by VR (Valine-Arginine) GR (Glycine-Arginine), SR (Serine-Arginine), in which first residue shuffled from a non-polar to a polar residue and the second residue was mostly a positively charged residue R (Arginine). The third “x” existed in two forms (Isoleucine/Leucine), both having a non-polar nature. In the “Cordycipitaceae/Clavicipitaceae” clade, the first two “xx” were represented by DQ (Aspartate- Glutamine), RA (Arginine- Alanine), GR (Glycine-Arginine), AR (Alanine-Arginine), SR (Serine-Arginine) and DR (Aspartate-Arginine), in which the first residue varied from negatively charged to a non-polar/polar residue; the second residue which was an almost conserved R (Arginine), was replaced by Q (Glutamine) in only one member; the third “x” changed from non-polar residue V (Valine) to another non-polar residue I (Isoleucine). In the Nectriaceae clade, the first two “xx” were occupied by GT (Glycine-Threonine) or AS (Alanine-Serine) residues, in which the first was a non-polar and second was a polar amino acid residue. The third “x” position was represented by V/I/L (Valine, Isoleucine, Leucine) in a manner similar to the “Ophiocordycipitaceae” and “Cordycipitaceae/ Clavicipitaceae” clades.

Motif M2 (HxxFxGRA) was also detected in the mid region of the protein sequences of Proteinase K (S08.054). This motif was highly conserved within the 3 clades; however, the variable amino acid positions (x) were conserved in a clade specific manner. The first “x” position varied from a non-polar to polar residue and the second “x” position was occupied by a negatively charged amino acid in both the “Ophiocordycipitaceae” and “Cordycipitaceae/Clavicipitaceae” clades. In “Nectriaceae” the first “x” position was shared by N/K/S (polar or positively charged residues) and second “x” was E, a negatively charged amino acid similar to the other two clades. The chemical nature of the third “x” of this motif varied from negatively charged to non-polar/ polar residue. It was occupied by E (Glutamate) in “Ophiocordycipitaceae” clade, E/G (Glutamate/Glycine) in “Cordycipitaceae/Clavicipitaceae” clade, and E/Q/G (Glutamate/Glutamine/Glycine) in “Nectriaceae” clade.

Motif M4 DxxAPG was situated towards the C-terminal of Proteinase K protein. This motif was highly conserved among all 58 sequences of proteinase K (S08.054) family. In this motif the two “xx” positions were also quite conserved in their chemical nature and were occupied by a non-polar residue and a polar residue respectively among all three clades.

Considerable conservation of motifs between the protein sequences and variability in residues in a clade specific manner observed in motif analyses proposed that the variation in the conserved residues could play a significant role in imparting clade specific differences in the enzymatic activity and stability of Subtilisin (S08.005) and Proteinase K (S08.054) protein families.

### Positive selection in protein sequence and biological significance

Selection pressure helps evolve proteins to acquire function according to the environmental conditions. Positive selection promotes the fixation of beneficial mutations in a population and leads to functional shift of a protein [32]. The ratio of nonsynonymous substitutions per nonsynonymous site (dN) to synonymous substitutions per synonymous site (dS) is termed as ω, which measures selective pressure on a sequence. If ω > 1 it signifies positive selection pressure, ω = 1 signifies neutral evolution, while ω < 1 indicates purifying selection pressure.

Site models and branch-site models in CODEML were used to detect positive selection along the pre-specified groups. These models test at the codon level if a hypothesis which allows for positive selection (models M2a and M8) is a better fit to the data than a null neutral hypothesis (models M1a and M7). Site-models identified sites that are under recurrent positive selection across the phylogenetic tree. Branch-site models detected sites that have been under positive selection at a particular point of evolution, i.e. on a specific branch of the evolutionary tree.

### Analysis of paralogous clades of Subtilisin (S08.005) proteins based on Site and Branch-site models

Site models and Branch-site models based analysis carried out for estimation of selective pressure on the Subtilisin (S08.005) gene family in Hypocreales is presented in Tables 1 and 2 respectively. After removal of gaps a total of 600 sites were tested for positive selection by using CODEML program. The site models allow the *ω* ratio to vary among codons. The LRTs were significant in M1a vs. M2a and M7 vs. M8 comparisons. Our results suggested the following: (i) Model M2a fits the data better and (ii) positive selection prevailed over neutral selection when M1a and M2a models were compared. In M2a analysis, four sites were detected as positively selected sites with a *p*-value < 0.05 (Table 1).

**Table 1:** Likelihood estimates of Subtilisin (S08.005) gene family for site models in PAML

Model	np	Parameter estimates (Proportion p, omega ω)	lnL	LRT pairs	df	2ΔlnL	*P*	Positively selected sites (BEB)
site model								
M0:one ratio	106	*ω* = 0.13223	−28649.08	M0/M3	4	0	1	
Model M3:Discrete	110	*p*_*0*_ = 0.00000	−28649.08					
		*p*_1_ = 0.00000						
		*p*_2_ = 1.00000						
		ω _0_ = 0.00000						
		ω_1_ = 0.00000						
		ω_2_ = 0.13223						
M1a(neutral)	107	*p*_*0*_ = 0.00001	−30002.75	M1a/M2a	2	86.44	**0.00**	
		*p*_1_ = 0.99999						
		ω_0_ = 0.00001						
		ω_1_ = 1.00000						
Model 2a: Positive Selection	109	*p*_*0*_ = 0.00000	−29959.53					4D*,5Q*,160S*,187G*
		*p*_1_ = 0.87583						
		*p*_2_ = 0.12417,						
		ω _0_ = 0.00000						
		ω_1_ = 1.00000						
		**ω**_**2**_ **= 2.09472**						
Model 7: beta	107	*p* = 2.04966	−28245.05	M7/M8	2	3515.4	**0.00**	
		*q* = 9.83402						
Model 8 :	109	*p*_*0*_ = 0.00001	−30002.75					
		*p* = 0.00500						
		*q* = 1.23069						
		*p*_1_ = 0.99999						
		ω = 1.00000						

Selection analysis by site models. np: number of free parameters. lnL: log likelihood. LRT: likelihood ratio test. df: degrees of freedom. 2∆lnL: twice the log-likelihood difference of the models compared. The significant tests at 95 % cut off are labeled with*. Bold: *P* < 0.05

**Table 2:** Likelihood estimates of Subtilisin (S08.005) gene family for branch-site models in PAML

Branch-Site (Model)	np	Parameter estimates (Proportion p, omega ω)	lnL	LRT Pairs	2ΔlnL	df	*P*	Positively selected sites (BEB)
Nectriaceae (BS_fix ω =1_)	108	*p*_*0*_ *=* 0.19, p_1_ = 0.04, p_2a_ = 0.62, p_2b_ = 0.14 ω_0=_0.15, ω_1_ = 1.00 b:ω_2a_ = 0.15, ω_2b_ = 1.00 f: ω_2a_ = 1.00, ω_2b_ = 1.00	−28503.14	BS_fix ω =1_/ BS_fix ω =0_	0	1	1	
Nectriaceae (BS_fix ω =0_)	109	*p*_*0*_ *=* 0.199, p_1_ = 0.04, p_2a_ 0.62, p_2b_ = 0.14, ω_0=_ 0.15, ω_1_ = 1.00, b:ω_2a_ = 0.15, ω_2b_ = 1.00 f: ω_2a_ = 1.00, ω_2b_ = 1.00	−28503.14					
Cordycipitaceae /Clavicipitaceae (BS_fix ω =1_)	108	*p*_*0*_ *=* 0, p_1_ = 0, p_2a_ =0.81, p_2b_ = 0.18 ω_0=_ 0.15, ω_1_ = 1.00, b:ω_2a_ = 0.15, ω_2b_ = 1.00 f: ω_2a_ = 1.00	−28503.00	BS_fix ω =1_/BS_fix ω =0_	0.034	1	0.85	
Cordycipitaceae/Clavicipitaceae (BS_fix ω =0_)	109	*p*_*0*_ *=* 0.00, p_1_ = 0.00, p2a =0.81, p_2b_ = 0.18 ω_0=_ 0.15, ω_1_ = 1.00, b:ω_2a_ = 0.15, ω_2b_ = 0.15 **f: ω**_**2a**_ **= 999.00, ω**_**2b**_ **= 999.00**	−28502.99					
Ophiocordycipitaceae (BS_fix ω =1_)	108	*p*_*0*_ = 0.27, p_1_ = 0.06,p_2a_ =0.54, p_2b_ = 0.12 ω_0_ = 0.15, ω_1_ = 1.00, b:ω_2a_ = 1.00, ω_2b_ = 1.00 f:ω_2a_ = 1.00,ω_2b_ = 1.00	−28501.50	BS_fix ω =1_/BS_fix ω =0_	10.64	1	**0.001**	
Ophiocordycipitaceae (BS_fix ω =0_)	109	*p*_*0*_ = 0.78*,*p_1_ = 0.18,p_2a_ =0.03, p_2b_ = 0.00 ω_0=_ 0.15, ω_1_ = 1.00, b:ω_2a_ = 1.00, ω_2b_ = 1.00 f: **ω**_**2a**_ **= 999.00,ω**_**2b**_ **= 999.00**	−28496.18					

Selection analysis by branch-site models. BS: branch-site. lnL: log likelihood. LRT: likelihood ratio test. df: degrees of freedom. 2∆lnL: twice the log-likelihood difference of the models compared. Bold: *P* < 0.05

In order to identify occurrence of positive selection in specific stages of evolution or in specific branches, branch site models were employed. Positively selected amino acid sites in three branches/clades (Nectriaceae, Cordycipitaceae/Clavicipitaceae and Ophiocordycipitaceae) were searched. We found very high dN/dS ratio (f: ω_2a=_999.00, ω_2b=_ 999.00) (Table 2), which indicated that gene sequences were positively selected in the “Cordycipitaceae/Clavicipitaceae” and “Ophiocordycipitaceae” branches.

### Analysis of paralogous clades of Proteinase K (S08.054) based on Site and Branch-site models

Site models and Branch-site models based analyses carried out for estimation of selection pressure on the Proteinase K (S08.054) gene family in Hypocreales is presented in Tables 3 and 4. After removal of gaps a total of 435 sites were analyzed using the CODEML program. The LRT value was found significant for M0 vs. M3 model only. The results indicated that M3 fits the data better which indicated variable selection pressure (evolutionary rate heterogeneity) among sites.

**Table 3:** Likelihood estimates of Proteinase K (S08.054) gene family for site models in PAML

Model	np	Parameter estimates (Proportion p, omega ω)	lnL	LRT pairs	Df	2ΔlnL	*P*	Positively selected sites (BEB)
site model								
M0:one ratio	116	*ω* = 0.12397	−19267.73	M0/M3	4	1537	**0.00**	
Model M3:Discrete	120	*P*_*0*_ = 0.23156	−18499.23					
		*p*_1_ = 0.41651						
		*p*_2_ = 0.35193						
		ω_0_ = 0.01542						
		ω_1_ = 0.08491						
		ω_2_ = 0.31104						
M1a(neutral)	117	*p*_*0*_ = 0.66046	−18857.30	M1a/M2a	2	0	1	
		*p*_1_ = 0.33954						
		ω_0_ = 0.12388						
		ω_1_ = 1.00000						
Model 2a: Positive Selection	119	*p*_*0*_ = 0.66046	−18857.30					
		*p*_1_ = 0.09285						
		*p*_2_ = 0.24669						
		ω_0_ = 0.12388						
		ω_1_ = 1.00000						
		ω_2_ = 1.00000						
Model 7: beta	117	*p* =0.81898	−18473.64	M7/M8	2	0	1	
		*q* =4.31422						
Model 8 :	119	*p*_*0*_ = 0.99999	−18473.64					
		*p* = 0.81899						
		*q* =4.31430						
		*p*_1_ = 0.00001						
		ω = 1.00000						

Selection analysis by site models. np: number of free parameters. lnL: log likelihood. LRT: likelihood ratio test. df: degrees of freedom. 2∆lnL: twice the log-likelihood difference of the models compared. Bold: *P* < 0.05

**Table 4:** Likelihood estimates of Proteinase K (S08.054) gene family for branch-site models in PAML

Branch- Site (Model)	np	Parameter estimates (Proportion p, omega ω)	lnL	LRT Pairs	2Δ lnL	df	*P*	Positively selected sites (BEB)
Nectriaceae (BS_fix ω =1_)	118	*p*_*0*_ *= 0.65*,p_1_ = 0.33,p_2a_ = 0.003,p_2b_ = 0.001 ω_0=_ 0.123, ω_1_ = 1.00b:ω_2a_ = 0.123, ω_2b_ = 1.000 f: ω_2a_ = 1.00, ω_2b_ = 1.00	−18857.30	BS_fix ω =1_/ BS_fix ω =0_	0	1	1	
Nectriaceae (BS_fix ω =0_)	119	*p*_*0*_ *= 0.39*,p_1_ = 0.20,p_2a=_0.27, p_2b_ = 0.13,ω_0_ = 0.123,ω_1_ = 1.00, b:ω_2a_ = 0.123,ω_2b_ = 1.00 f: ω_2a_ = 2.09, ω_2b_ = 2.09	−18857.30					
Cordycipitaceae/Clavicipitaceae (BS_fix ω =1_)	118	*p*_*0*_ *=* 0.66, p_1_ = 0.33, p_2a_ = 0.00,p_2b_ = 0.00 ω_0=_0.123 ,ω_1_ = 1.00, b:ω_2a_ = 0.123, ω_2b_ = 1.00 f: ω_2a_ = 1.00, ω_2b_ = 1.00	−18857.30	BS_fix ω =1_/BS_fix ω =0_	0	1	1	
Cordycipitaceae/Clavicipitaceae (BS_fix ω =0_)	119	*p*_*0*_ *=* 0.66,p_1_ = 0.33, p_2_a = 0.00,p_2b_ = 0.00 ω_0=_ 0.123 ,ω_1_ = 1.00,b:ω_2a_ = 0.123, ω_2b_ = 1.00 f: ω_2a_ = 1.00,ω_2b_ = 1.00	−18857.30					
Ophiocordycipitaceae (BS_fix ω =1_)	118	*p*_*0*_ = 0.38,p_1_ = 0.19,p_2a_ = 0.27,p_2b_ = 0.14 ω_0_ = 0.12102,ω_1_ = 1.00, b:ω_2a_ = 0.12, ω_2b_ = 1.00 f:ω_2a_ = 1.00,ω_2b_ = 1.00	−18849.02	BS_fix ω =1_/ BS_fix ω =0_	2.04	1	0.15	
Ophiocordycipitaceae (BS_fix ω =0_)	119	*p*_*0*_ = 0.53*,*p_1_ = 0.28,p_2a_ = 0.11,p_2b_ = 0.062 ω_0=_ 0.12 ,ω_1_ = 1.00,b:ω_2a_ = 0.12,ω_2b_ = 1.00 **f: ω**_**2a**_ **= 5.11,ω**_**2b**_ **= 5.11**	−18848.00					69 S*, 103 I*

Selection analysis by branch-site models. BS: branch-site. lnL: log likelihood. LRT: likelihood ratio test. df: degrees of freedom. 2∆lnL: twice the log-likelihood difference of the models compared. The significant tests at 95 % cut off are labeled with*. Bold: *P* < 0.05

To detect specific stages of evolution and positive selection in Proteinase K gene family, branch site models were employed to search for amino acid sites under positive selection in branches for 3 clade branch sites: “Nectriaceae”, “Cordycipitaceae/Clavicipitaceae” and “Ophiocordycipitaceae”. In the “Ophiocordycipitaceae” clade, two positively selected sites (69 S*, 103 I*) were detected with a p-value <0.05. This observation is in accordance with our findings for the Subtilisin (S08.005) gene family. Our results suggested that “Ophiocordycipitaceae” clade consisting of protein sequences exclusively of *P. lilacinum* recorded maximum sites under positive selection in the evolutionary tree. Subtilisin-like proteins are known to play crucial role in trapping and pathogenesis of nematode in nematophagous fungi [12, 21–23]. The observed positive selection on protein residues and functional shift in protein sequences could have enabled successful pathogen-host interaction in *P. lilacinum.*

### Analysis of type I and type II functional divergence

Protein residues could be subjected to altered functional constraints during evolution especially after gene duplication [33]. Evaluation of functional constraints operating on amino acid residues of Subtilisin (S08.005) and Proteinase K (S08.054) gene families was carried out by DIVERGE (version 3.0). DIVERGE analysis tests for the presence of functional divergence of two types, type I and type II. Functional divergence type I stands for significant variability between the duplicate genes in paralogous clades at conserved sites. Type I value indicates the selection pressure at particular protein residue site, due to the acquisition or pre-existence of a functional role for that site, in one of the clades compared to the paralogous clade. Functional divergence type II identifies the mutations that after gene duplication lead to fixation of different amino acids in the paralogous clades. These mutations remain conserved after speciation in each clade.

### Functional divergence in Subtilisin (S08.005) gene family

Clades “Ophiocordycipitaceae”, “Nectriaceae” and “Cordycipitaceae/Clavicipitaceae” which consisted of proteins from specific families in the phylogenetic analysis, were examined for functional divergence in a pair-wise manner. The log-likelihood values for functional divergence type I analysis supported hypothesis of existence of functional divergence than the null hypothesis of no functional divergence: “Nectriaceae” vs. “Cordycipitaceae/Clavicipitaceae” (θ = 0.999 ± 0.079; LRT = 156.42); “Nectriaceae” vs. “Ophiocordycipitaceae” (θ = 0.99 ± 0.07; LRT = 175.88). 166 distinct type 1 divergence sites were observed which are conserved in “Nectriaceae” clade but showed divergence (operating under selection pressure) in the other two clades. Type II analysis was highly significant with a value of θII = 0.65 ± 0.07 (“Nectriaceae” vs. “Cordycipitaceae/Clavicipitaceae”) and revealed 109 putative divergent sites at threshold posterior ratio (R) of 2.03; θII = 0.58 ± 0.07 (“Nectriaceae” vs. “Ophiocordycipitaceae”) and revealed 102 putative divergent sites at threshold posterior ratio (R) of 1.87. The values of divergence coefficient for type I and type II analyses (θI and θII >0) for all pairs are presented in (Table 5). For all the above mentioned subsets, the values of θI and θII were greater than 0, which suggested that strong functional divergence signals could be picked up between these clades. The significant divergence values implied occurrence of site-specific altered selective constraints/ radical shifts in amino acid physiochemical properties following gene duplication and/or speciation. Detailed analysis of these sites would help in delineating the amino acids that are responsible for differences in biochemical features and structural stability in Subtilisin (S08.005) proteins in these clades (Additional file 7: Table S2).

**Table 5:** Divergence analysis among Subtilisin (S08.005) genes in Hypocreales. Functional divergence estimates of type I and type II of two clusters comparison are shown

	Functional Divergence type I		
Clades	Nectriaceae vs. Cordycipitaceae/Clavicipitaceae	Nectriaceae vs. Ophiocordycipitaceae	Ophiocordycipitaceae vs. Cordycipitaceae/ Clavicipitaceae
Subtilisin (S08.005)			
θml	0.9992	0.9992	0.1432
SE θ	0.079892	0.075343	0.069332
LRT θ	156.423961	175.881442	4.266
Cut off *p*-value	0.99	0.99	0.50
Sites	166 sites	166 sites	2 sites
Proteinase K (S08.054)	No Divergence		
	Functional Divergence type II		
Clades	Nectriaceae vs. Cordycipitaceae/ Clavicipitaceae	Nectriaceae vs. Ophiocordycipitaceae	Ophiocordycipitaceae vs. Cordycipitaceae/Clavicipitaceae
Subtilisin (S08.005)			
^a^C	41	45	29
^b^R	68	57	48
θII	0.64815	0.576089	−0.162629
SE θ	0.07596	0.074653	0.286306
^c^Ar	0.630576	0.58723	−0.380073
^d^Plr	0.31213	0.31213	0.31213
Posterior Ratio (R)	2.03	1.87	−1.21
Sites	109 sites	102 sites	No Divergence

^a^C: Number of sites with conserved change between two clusters

^b^ R: Number of sites with radical change between two clusters

^c^Ar: Proportion of radical changes under F2-state (type-II functional divergence)

^d^PIr: Proportion of radical (πR) changes under F0-state (no functional divergence)

Variation in evolutionary rates among residues (RVS) within a given protein is partially attributable to positive diversifying selection leading to adaptation to environmental changes. Site specific evolutionary rates are suggested to be governed by interplay between structural and functional constraints [34]. We observed that 16 out of 19, 48 out of 59 and 50 out of 62 RVS sites in “Nectriaceae”, “Cordycipitaceae/Clavicipitaceae” and “Ophiocordycipitaceae” clades respectively also experienced type II divergence (Additional file 7: Table S2, Additional file 8: Figure S6). The overlap between the RVS and type II sites highlighted the determining contribution of these residues in protein function and evolution.

### Functional divergence in Proteinase K (S08.054) gene family

No functional divergence between the paralogous clades among protein sequences of the Proteinase K (S08.054) family was observed (Table 5) and therefore, the sequences were not included for further analyses in the study.

### 3D structure modelling of protein sequences and mapping of important residues observed in DIVERGE analysis

**Fig. 7:**
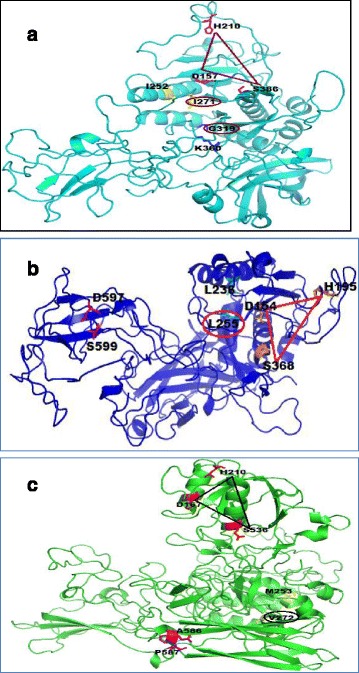
Functional divergence in Subtilisin (S08.005) protein sequences. **a** Subtilisin structure of a member (fx|XP5977|*Fusarium oxysporum*) of the “Nectriaceae” clade is shown. The amino acid residues that experienced site-specific rate shift (RVS) and/or type II divergence and fell into the catalytic triad/active sites/substrate binding sites of the enzyme are highlighted. The catalytic triad is shown by a triangle (brown colour). Predicted active sites are shown in yellow colour; substrate binding sites are shown in blue colour. Amino acids highlighted in brown colour experienced type-II divergence and was present in an active site and a binding site. **b** Subtilisin structure of a member (bb|XP2612|*Beauveria bassiana*) of the “Cordycipitaceae/Clavicipitaceae” clade is shown. The amino acids residues that experienced site-specific rate shift (RVS) and/or type II divergence and fell into the catalytic triad/active sites/substrate binding sites of the enzyme are highlighted. Predicted catalytic triad is shown by a triangle (red in colour); Active sites in cyan colour, Substrate binding sites in magenta colour. Amino acid highlighted in red experienced both RVS and type-II divergence and was present in one of the active sites. **c** Subtilisin structure of a member (pl|XP5939|*Purpureocillium lilacinum*) of the “Ophiocordycipitaceae” clade is shown. The amino acids residues that experienced site-specific rate shift (RVS) and/or type II divergence and fell into the catalytic triad/active sites/substrate binding sites of the enzyme are highlighted. Predicted catalytic triad is shown by a triangle (black in colour); Active sites in yellow colour, Substrate binding sites in magenta colour. Amino acid highlighted in black experienced type-II divergence and was present in one of the active sites

To analyse the possible role of amino acids identified under DIVERGE analyses on the function and structure of Subtilisin (S08.005) proteins, the type II and RVS (rate variation among sites) sites were mapped on the secondary structure of the proteins (Additional file 8: Figure S6, S7, S8). A total of 7, 6 and 4 type II divergence sites and 0, 5 and 4 RVS sites in “Nectriaceae”, “Cordycipitaceae/Clavicipitaceae” and “Ophiocordycipitaceae” clades respectively are part of alpha helices (Additional file 7: Table S2) that constitute the backbone peptide bonds of the protein. Presence of particular RVS and type II divergence sites on secondary structure of the proteins argued for their putative involvement in protein structure stability/alteration and evolution. I271 and G319 residues that experienced type II divergence were part of the predicted active and binding sites of the protein in “Nectriaceae” clade; L255 residue that experienced type II divergence and RVS was located at the predicted active site of the protein in “Cordycipitaceae/Clavicipitaceae” clade; V272 residue experienced type II divergence and was part of the predicted active site of the protein in “Ophiocordycipitaceae” clade (Fig. 7). These findings indicated that there is substantial contribution of divergent sites in defining the catalytic triad, active site and substrate binding cavity of the Subtilisin (S08.005) proteins of the three paralogous clades and putatively helped in functional shift of these proteins among species of Hypocreales.

## Conclusion

Subtilases seemed to be major determinants of adaptation in Hypocrealean fungi according to the changing environment, lifestyle and host. Phylogenetic analysis and identification of amino acid residues under positive selection and type II divergence provided insights into specific adaptation mechanisms. RVS and type II sites identified on the secondary structure, catalytic triads, active sites and substrate binding sites of the Subtilisin (S08.005) proteins of the three paralogous clades could be responsible for functional shift of these proteins during the course of evolution.

## Methods

### Identification of Subtilase (Subtilisin (S08.005), Proteinase K (S08.054) and Serine-carboxyl peptidase (S53.001) genes

Subtilase gene sequences of 10 fungal species distributed in 5 families under the order Hypocreales were identified by MEROPS [http://merops.sanger.ac.uk/] [24] analysis of WGS assemblies available in NCBI. Whole genome nucleotide and protein sequences of *F. oxysporum* (http://www.ncbi.nlm.nih.gov/bioproject/18813), *F. graminearum* (http://www.ncbi.nlm.nih.gov/bioproject/PRJNA235346), *T. reesei* (http://www.ncbi.nlm.nih.gov/bioproject/PRJNA266930), *B. bassiana* (http://www.ncbi.nlm.nih.gov/bioproject/38719), *C. militaris* ( http://www.ncbi.nlm.nih.gov/bioproject/PRJNA41129/), *M. robertsii* (http://www.ncbi.nlm.nih.gov/bioproject/PRJNA245140/), *M. acridum* (http://www.ncbi.nlm.nih.gov/bioproject/38715/), *P. chlamydosporia* ( http://www.ncbi.nlm.nih.gov/bioproject/68669/) and *T. inflatum* ( http://www.ncbi.nlm.nih.gov/bioproject/PRJNA73163/), were downloaded from NCBI database (http://www.ncbi.nlm.nih.gov/). Structural annotation of BioProjects of *P. chlamydosporia* and *T. inflatum* was carried out using Augustus tool [35] to predict respective gene and protein sequences.

### Phylogenetic analyses

The multiple alignment of full-length protein sequences of 53 Subtilisin (S08.005), 58 Proteinase K (S08.054) and 50 Serine-carboxyl peptidase (S53.001) genes were performed using MUSCLE (Multiple Sequence Comparison by Log-Expectation) [36, 37]. The profiles of the created alignment protein sequences were used to construct three separate maximum likelihood (ML) phylogenetic trees by MEGA 6.0 [38] for Subtilisin (S08.005), Proteinase K (S08.054) and Serine-carboxyl peptidase (S53.001) genes families respectively. The support to the interior branches and clades of the phylogenetic trees was estimated through 1000-iteration bootstrap resampling.

### Estimating gene gain and loss via gene tree/species tree reconciliation

Subtilisin gene (S08.005) tree (Fig. 1) and Proteinase K gene (S08.054) tree (Fig. 2) were compared with the rooted species tree [4] to map each node in the gene tree as either a speciation or a duplication event. NOTUNG (version 2.8) [30] was used for the reconciliation of gene tree and species tree. NOTUNG is based on the maximal parsimony method and outputs the reconciled tree that minimizes the overall duplication/loss. NOTUNG was performed with the 90 % (default) bootstrap cutoffs to collapse poorly supported topologies. By reconciliation of the gene tree and species tree using different modes of NOTUNG, the number of gene gain and losses were determined (Figs. 3 and 4, Additional file 3: Figure S2 and Additional file 4: Figure S3).

### Motif prediction

Gene alignments were analyzed by the MEME program (http://meme.sdsc.edu) [31] for the prediction of conserved motifs. MEME was run with the following parameters: number of repetitions = one occurrence per sequence, maximum number of motifs = 5, and optimum motif width was constrained to between 5 and 15 residues.

### Estimating the pattern of nucleotide substitution and positive selection sites

Positive selection on Subtilisin (S08.005) gene family during evolution was determined by applying a maximum-likelihood approach in the CODEML program of PAML v4.8 (http://abacus.gene.ucl.ac.uk/software/paml.html) [39]. Codon-based likelihood methods, site models and branch-site models, were performed. Maximum likelihood estimations of selection pressure were based on the ratio (ω) of the nonsynonymous (dN) and synonymous substitution rates (dS), dN/dS [40]. The parameter estimates (ω) and likelihood scores were calculated for three pairs of models: M0 (one ratio) versus M3 (discrete), M1a (nearly neutral) versus M2a (positive selection) and M7 (beta) versus M8 (beta + ω). The likelihood ratio test (LRT) was used to compare two nested models and twice the log likelihood difference between the two models (2ΔL) was assumed to follow a χ2 distribution with degrees of freedom equal to the difference in the number of free parameters in the test. *P* <0.05 was considered significant. Bayes empirical Bayes (BEB) method was employed to identify sites under positive selection, neutral or purifying selection in the foreground group with significant LRTs.

### Estimation of functional divergence

DIVERGE (DetectIng Variability in Evolutionary Rates among GEnes) v3.0 program (http://xgu1.zool.iastate.edu) [33] was employed for estimation of type I and type II functional divergence between the gene clusters of the Subtilisin (S08.005) & Proteinase K (S08.054) families through posterior analysis. The coefficients of type I and type II functional divergence (θI and θII) between members of all pairs of interesting clades were calculated [33, 41–44]. Values of θI and θII significantly greater than 0, implied site-specific altered selective constraints or radical shifts in amino acid physiochemical properties following gene duplication and/or speciation. Large Qk values indicated a high probability that evolutionary rates, or site-level physiochemical amino acid properties, differed between two clades.

### Three dimensional structure prediction of Subtilisin (S08.005) proteins

For the structural analysis of Subtilisin (S08.005) proteins, a representative protein sequence from each of three paralogous clades was selected based on the identification of their homolog in pathogen-host interaction (PHI) database (http://www.phi-base.org/) [45]. HHpred (http://toolkit.tuebingen.mpg.de/hhpred) [46, 47] method was used to find out the suitable template based on homology detection. The Subtilisin (S08.005) sequences shared low sequence similarity with the known structures. Therefore, the sequences of Subtilisin (S08.005) from “Nectriaceae”, “Cordycipitaceae/Clavicipitaceae”, and “Ophiocordycipitaceae” clades were modeled using fold recognition method through Phyre2 server (http://www.sbg.bio.ic.ac.uk/phyre2/html) [48].

## Additional files

Additional file 1: Table S1. Accession IDs of protein sequences included in the phylogenetic analysis (XLSX 16 kb)
Additional file 2: Figure S1. Phylogenetic relationships among protein sequences belonging to the Serine-carboxyl peptidases (S53.001) family. The numbers indicate the Bootstrap values for each branch. (DOCX 37 kb)
Additional file 3: Figure S2. Gene tree of Subtilisin (S08.005) predicted by NOTUNG (rearrange mode). (DOCX 61 kb)
Additional file 4: Figure S3. Gene tree of Proteinase K (S08.054) predicted by NOTUNG (rearrange mode). (DOCX 53 kb)
Additional file 5: Figure S4. Conserved Motifs identified by MEME in Subtilisin (S08.005). (DOCX 52 kb)
Additional file 6: Figure S5. Conserved Motifs identified by MEME in Proteinase K (S08.054). (DOCX 36 kb)
Additional file 7: Table S2. Functional Divergence in Subtilisin (S08.005). (XLSX 39 kb)
Additional file 8: Figure S6. Functional divergence in Subtilisin (S08.005) protein sequences among clades Nectriaceae and Cordycipitaceae/Clavicipitaceae. (a) The RVS (Rate Variation among Sites) amino acids sites are identified by DIVERGE 3.0 mapped on the Subtilisin structure (Cyan colour) of a member (fx|XP5977|*Fusarium oxysporum*) of the Nectriaceae clade. The identified RVS sites are shown in stick (Magenta). (b) The highlighted (encircled) **RVS sites** are also observed in TYPE II divergence (Nectriaceae vs. Cordycipitaceae/Clavicipitaceae). **Figure S7.** Functional divergence type II and RVS sites on 3D structure of Subtilisin (S08.005) protein sequences among clades Nectriaceae and Cordycipitaceae/Clavicipitaceae. (a) The RVS (Rate Variation among Sites) amino acids sites are identified by DIVERGE 3.0 mapped on Subtilisin structure (Red colour) of a member (bb|XP2612|*Beauveria bassiana*) of the Cordycipitaceae/Clavicipitaceae clade. The identified RVS sites are shown in stick (Green). (b) The highlighted **RVS sites** are also observed in TYPE II divergence (Nectriaceae vs. Cordycipitaceae/Clavicipitaceae). The catalytic triad is shown in yellow colour. **Figure S8.** Functional divergence in Subtilisin (S08.005) when Nectriaceae and Ophiocordycipitaceae clades are compared. (a) The RVS (Rate Variation among Sites) amino acids sites are identified by DIVERGE 3.0 mapped on Subtilisin structure (Blue colour) of a member (pl|XP5939|*Purpureocillium lilacinum*) of the Ophiocordycipitaceae clade. The identified RVS sites are shown in stick (Yellow). (b) The highlighted (encircled) **RVS sites** are also observed in TYPE II divergence (Nectriaceae vs. Ophiocordycipitaceae). The putative catalytic triad and substrate binding pocket are shown in blue colour on the 3 D structure of the protein. (DOCX 844 kb)

## Abbreviations

*B. bassiana*: *Beauveria bassiana*
BEB: Bayes empirical Bayes
*C. militaris*: *Cordyceps militaris*
df: Degree of freedom
DIVERGE: DetectIng variability in evolutionary rates among GEnes
*F. graminearum*: *Fusarium graminearum*
*F. oxysporum*: *Fusarium oxysporum*
lnL: Log likelihood
LRT: Likelihood ratio test
*M. acridum*: *Metarhizium acridum*
*M. robertsii*: *Metarhizium robertsii*
MEME: Multiple EM for Motif Elicitation
ML: Maximum likelihood
MUSCLE: Multiple sequence comparison by log-expectation
*P. chlamydosporia*: *Pochonia chlamydosporia*
*P. lilacinum*: *Purpureocillium lilacinum*
PHI: Pathogen-host interaction
RVS: Rate variation among sites
*T. album*: *Tritirachium album*
*T. inflatum*: *Tolypocladium inflatum*
*T. reesei*: *Trichoderma reesei*
WGS: Whole Genome sequencing.
