# Supplementary figures and images for: Phylogenetic analyses reveal molecular signatures associated with functional divergence among Subtilisin like Serine Proteases are linked to lifestyle transitions in Hypocreales
Source: BMC Evol Biol. 2016 Oct 19;16:220. doi: 10.1186/s12862-016-0793-y (PMC5069783; doi:10.1186/s12862-016-0793-y)

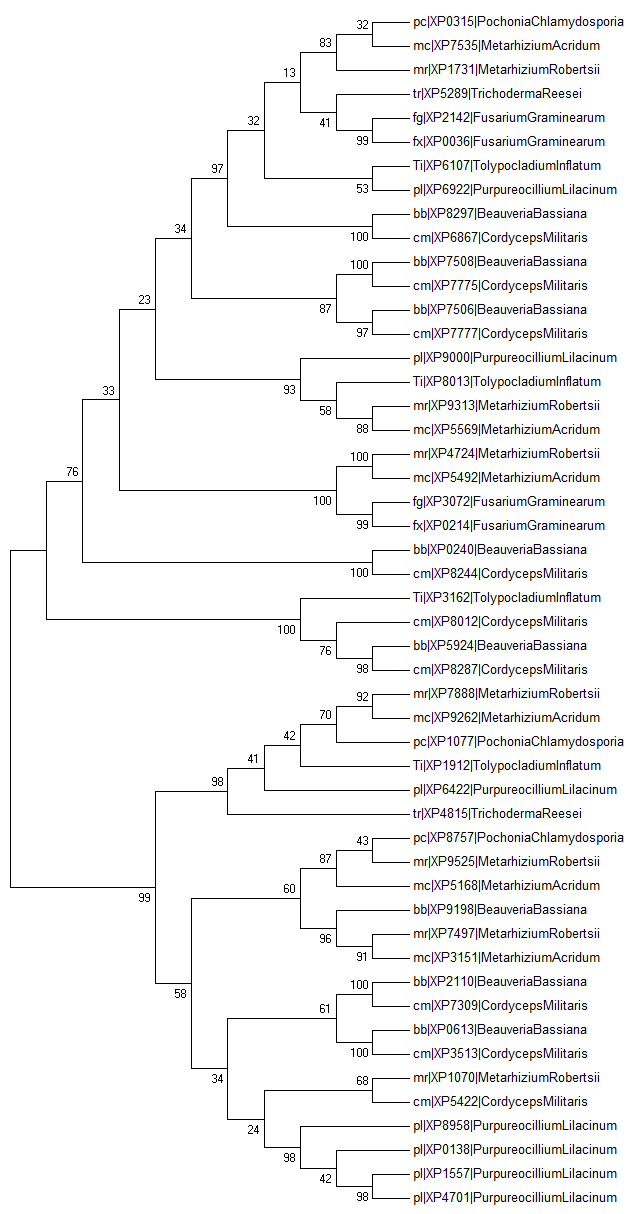


**Figure S1.**

Supplement: Additional file 2: Figure S1. — Phylogenetic relationships among protein sequences belonging to the Serine-carboxyl peptidases (S53.001) family. The numbers indicate the Bootstrap values for each branch. (DOCX 37 kb) [file 12862_2016_793_MOESM2_ESM.docx]

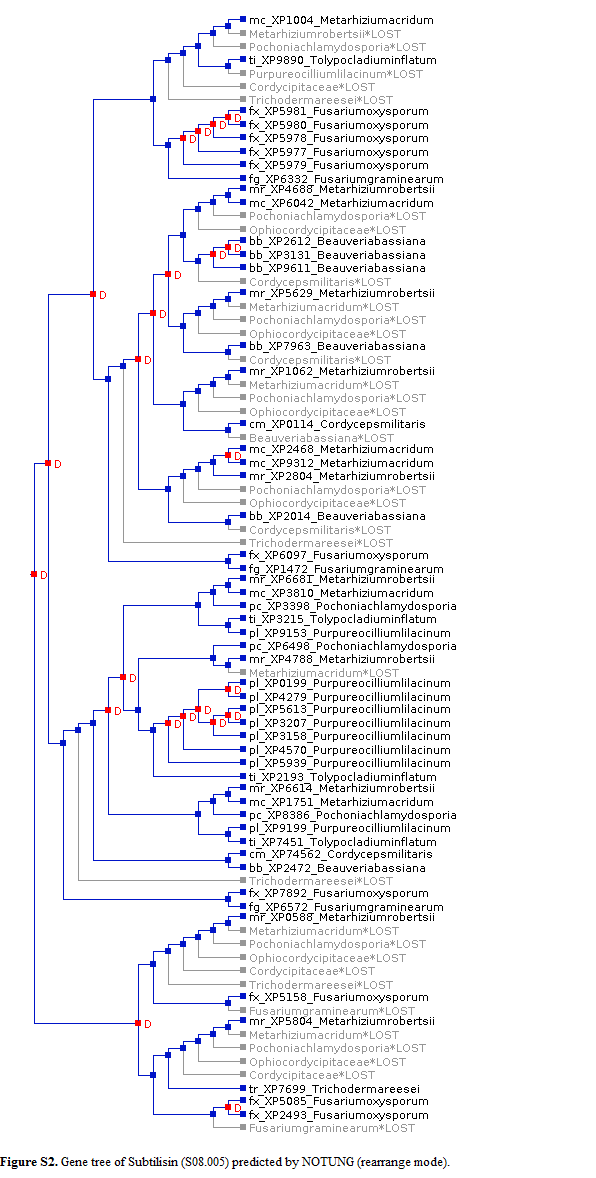


**FigureS2**

Supplement: Additional file 3: Figure S2. — Gene tree of Subtilisin (S08.005) predicted by NOTUNG (rearrange mode). (DOCX 61 kb) [file 12862_2016_793_MOESM3_ESM.docx]

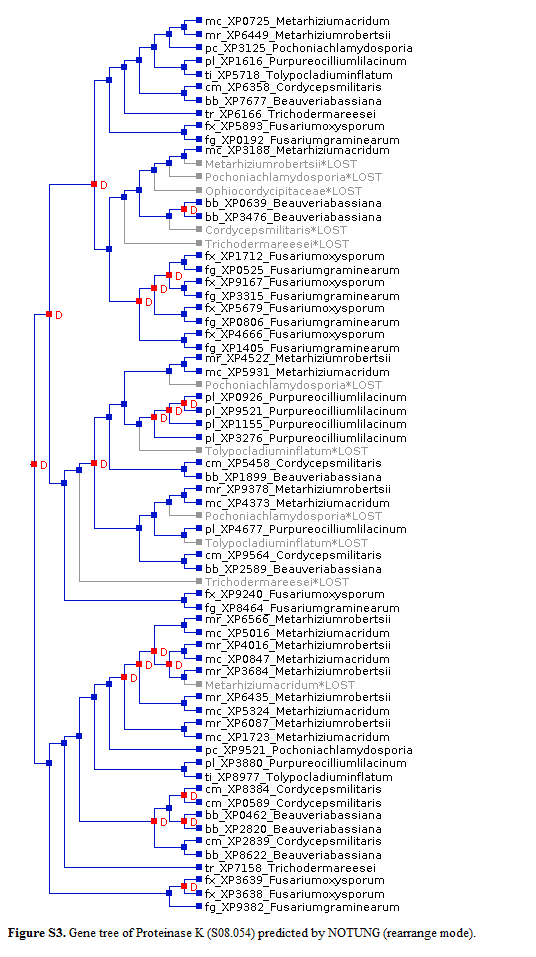


**FigureS3**

Supplement: Additional file 4: Figure S3. — Gene tree of Proteinase K (S08.054) predicted by NOTUNG (rearrange mode). (DOCX 53 kb) [file 12862_2016_793_MOESM4_ESM.docx]

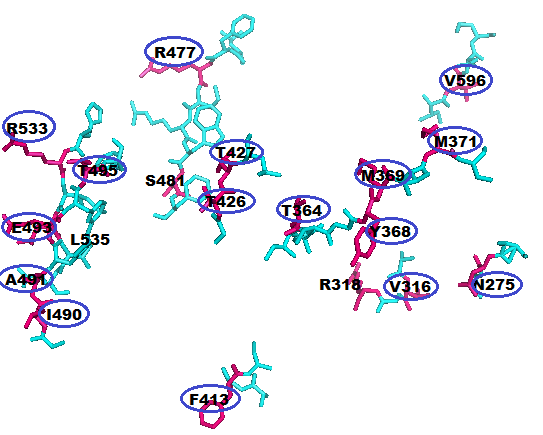

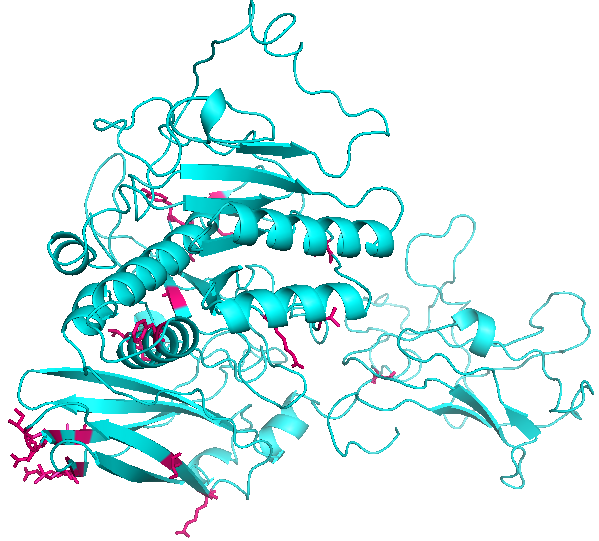


**(b)**

**(a)**

**Figure S6.**


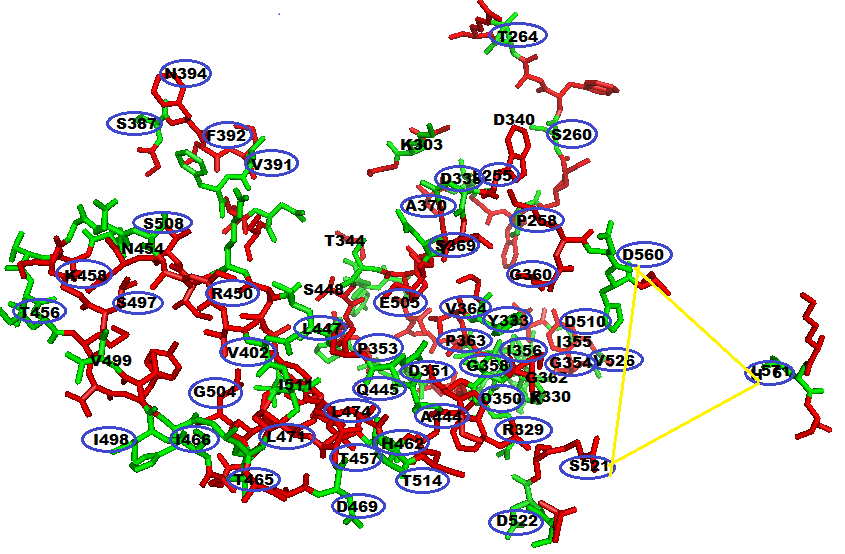


**(b)**

**(a)**


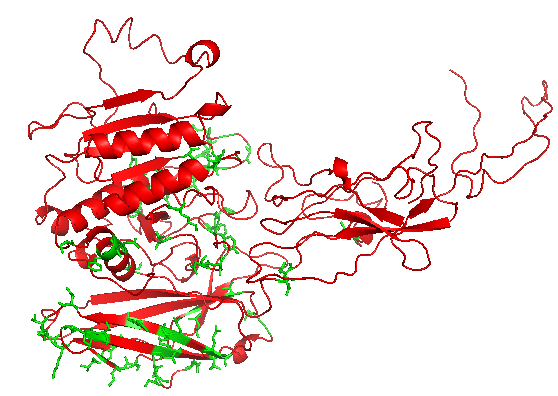


**Figure S7.**


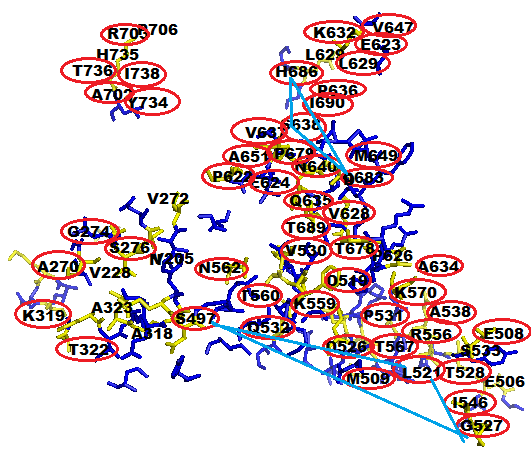

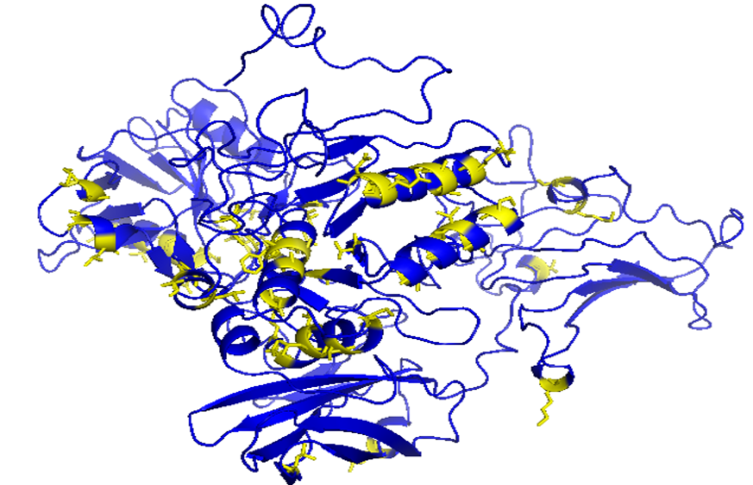


**(b)**

**(a)**

**FigureS8.**

Supplement: Additional file 8: Figure S6. — Functional divergence in Subtilisin (S08.005) protein sequences among clades Nectriaceae and Cordycipitaceae/Clavicipitaceae. (a) The RVS (Rate Variation among Sites) amino acids sites are identified by DIVERGE 3.0 mapped on the Subtilisin structure (Cyan colour) of a member (fx|XP5977|Fusarium oxysporum) of the Nectriaceae clade. The identified RVS sites are shown in stick (Magenta). (b) The highlighted (encircled) RVS sites are also observed in TYPE II divergence (Nectriaceae vs. Cordycipitaceae/Clavicipitaceae). Figure S7. Functional divergence type II and RVS sites on 3D structure of Subtilisin (S08.005) protein sequences among clades Nectriaceae and Cordycipitaceae/Clavicipitaceae. (a) The RVS (Rate Variation among Sites) amino acids sites are identified by DIVERGE 3.0 mapped on Subtilisin structure (Red colour) of a member (bb|XP2612|Beauveria bassiana) of the Cordycipitaceae/Clavicipitaceae clade. The identified RVS sites are shown in stick (Green). (b) The highlighted RVS sites are also observed in TYPE II divergence (Nectriaceae vs. Cordycipitaceae/Clavicipitaceae). The catalytic triad is shown in yellow colour. Figure S8. Functional divergence in Subtilisin (S08.005) when Nectriaceae and Ophiocordycipitaceae clades are compared. (a) The RVS (Rate Variation among Sites) amino acids sites are identified by DIVERGE 3.0 mapped on Subtilisin structure (Blue colour) of a member (pl|XP5939|Purpureocillium lilacinum) of the Ophiocordycipitaceae clade. The identified RVS sites are shown in stick (Yellow). (b) The highlighted (encircled) RVS sites are also observed in TYPE II divergence (Nectriaceae vs. Ophiocordycipitaceae). The putative catalytic triad and substrate binding pocket are shown in blue colour on the 3 D structure of the protein. (DOCX 844 kb) [file 12862_2016_793_MOESM8_ESM.docx]
