# Supplementary material for: Phylogenetic analyses reveal molecular signatures associated with functional divergence among Subtilisin like Serine Proteases are linked to lifestyle transitions in Hypocreales
Source: BMC Evol Biol. 2016 Oct 19;16:220. doi: 10.1186/s12862-016-0793-y (PMC5069783; doi:10.1186/s12862-016-0793-y)
Supplement: Additional file 5: Figure S4. — Conserved Motifs identified by MEME in Subtilisin (S08.005). (DOCX 52 kb) [file 12862_2016_793_MOESM5_ESM.docx]

**FigureS4**.

**MOTIF M1 (DKLxxG)**

fx|XP2493|FusariumOxysporum RW-----------------------------LRSIENFASAMAPYWEDTVTNFLETRQNS 806

fx|XP5158|FusariumOxysporum KW-----------------------------MKCMEDFSRRFKQIKA----------LRD 735

mr|XP0588|MetarhiziumRobertsii KW-----------------------------MQCMEEFASHFRQIRA----------LND 654

mr|XP5804|MetarhiziumRobertsii AW-----------------------------IKSTRQFATFLMNAS------------RE 748

fx|XP5085|FusariumOxysporum RW-----------------------------LNCMDKFADELQNTC------------SE 696

tr|XP7699|TrichodermaReesei ----------------------------------------------------------KV 238

ti|XP9890|TolypocladiumInflatum HPAFA-------------------------PQDHPMSYSMHRWTGV---------DKLHD 143

ma|XP1004|MetarhiziumAcridum LTNEI-------------------------NGNRPVNYSVHHWTGV---------DKLHE 94

fg|XP6332|FusariumGraminearum SKHG--------------------------PSSSIRNYSIHHWTGV---------DKLHA 95

fx|XP5981|FusariumOxysporum ------------------------------------------------------------ 0

fx|XP5977|FusariumOxysporum SESG--------------------------PSSSIRNYSIHHWTGV---------DKLHA 143

fx|XP5978|FusariumOxysporum SESG--------------------------PSSSIRNYSIHHWTGV---------DKLHA 143

fx|XP5979|FusariumOxysporum SESG--------------------------PSSSIRNYSIHHWTGV---------DKLHA 143

fx|XP5980|FusariumOxysporum SESG--------------------------PSSSIRNYSIHHWTGV---------DKLHA 143

bb|XP3131|BeauveriaBassiana T-------------------EH----PAAYQRRGNSAPWNHVMTQV---------DKLHA 171

ma|XP6042|MetarhiziumAcridum H-------------------HNHSTLL--QGLVAVKSGC-H-LGSI---------FVHTT 38

mr|XP4688|MetarhiziumRobertsii A-------------------ESQATPESAHQRRQVKSPWNHVMTQI---------DKLHA 142

bb|XP2612|BeauveriaBassiana A-------------------VP----EAGNQRRDTSSTWNHVMTQV---------DKLHA 140

bb|XP9611|BeauveriaBassiana P-------------------VQSPGQRSGPQRRDTSSSWNHIMTQI---------DKLHA 143

cm|XP0114|CordycepsMilitaris T-EM-EE--VKESLDR--RVRGSRGMK--ARAPETKAPWNHLMTQV---------DKLHA 159

mr|XP1062|MetarhiziumRobertsii GQEN-PK--LGAAGNL----HSGDTQRHRASKRAADDLWPHLMTHV---------DKLHK 149

bb|XP7963|BeauveriaBassiana PGEQ-PE--KQQSGHQ--QARWSSGLASRAAGQSLESPWHHVLTQV---------DKLHA 157

mr|XP5629|MetarhiziumRobertsii SL-------KPR-----------AGPKRQPYRRAVDTSWNHAMTQV---------DMLHS 141

ma|XP9312|MetarhiziumAcridum ------THATQQSFAGEKGQSLVERQALPGKNAAADTYAPHVMTQV---------DKLRD 167

bb|XP2014|BeauveriaBassiana SNDGAPKHEKHHSSIGKISKRQAMRHALRETN--TTAYPPHAMMQV---------DKLHR 159

ma|XP2468|MetarhiziumAcridum PSDR-AKGEKQRSFVGKLSKRQATRRALPETN--TTAYPPHVMMQV---------DKLRS 161

mr|XP2804|MetarhiziumRobertsii SSDR-PKSEKQESFVGKISKRRATRRALPETN--TTAYPPHVMMQV---------DKLRS 160

pl|XP0199|PurpureocilliumLilacinum CFVSDAS----A-ETIHANRADRPGLGAPDRSFEHNTFTPHVMTQV---------DKLHA 729

pl|XP9199|PurpureocilliumLilacinum NTNVIG---ALE-NIRT--------LGTHANTTEMAPFSPHVMTQV---------DKLHA 115

pl|XP5939|PurpureocilliumLilacinum ----GPSAG--------RSLTKRGALTARDSKSTAAALAPHIMAQV---------DRLHA 147

pl|XP4570|PurpureocilliumLilacinum ----L--QIIKA-KDLALNRDRHGLSARTNSSMHKDVFSTHVMTQV---------DKLHA 212

pl|XP3158|PurpureocilliumLilacinum ------GKVLTA-NDWNIKDGDRSQLSRRNIMNETDVWPPHIMTQV---------DKLRA 146

pl|XP3207|PurpureocilliumLilacinum ----N-GKPANL-KDMDFGGEADGDRLRRDVMNETDTWPPHVMTQV---------DKLRA 147

pl|XP5613|PurpureocilliumLilacinum ----ANSKALNI-KDLELGGKTDSALSRRDVMNETDTWPPHLMTQV---------EKLRA 148

pl|XP4279|PurpureocilliumLilacinum QVLSNPSGPVLV----------------NDTDDQDDIWSPHRMTQV---------DKLKA 149

pc|XP8386|PochoniaChlamydosporia DWNGNPSVGNNL-QAR-------------DASNR-APVSSHVMTQI---------DKLHA 138

ma|XP1751|MetarhiziumAcridum HWMGNPDGEKIL-QAR-------------DNSTLTNNFSPHFMTQI---------DKLHA 139

mr|XP6614|MetarhiziumRobertsii HWAGNPDREKIL-QAR-------------DNSTLTNNFSPHFMTQI---------DKLHA 139

pl|XP9153|PurpureocilliumLilacinum DWVGTPGETEQQ-ARKALHARQAGGGGGGFANMSGDAFSPHVMMQV---------DKLRA 154

ti|XP3215|TolypocladiumInflatum QWVGTPGMGTEG-NLRA---R---------GLNGTDTFSPHVMTQV---------DKLRA 140

bb|XP2472|BeauveriaBassiana EWVATQGLEALV-DKSGANI-------IDSRDTAEPISSAQRMGQI---------DKMRA 153

cm|XP74562|CordycepsMilitaris EWVATEGLQALV-HADP-SS-------INARDTAEPISSAQRMSQI---------EKMRA 152

ti|XP2193|TolypocladiumInflatum EWIGTNPREHFG-DVQR--R-------AP-ANETEDEFSPHVMTQV---------DQLRA 114

pc|XP3398|PochoniaChlamydosporia EWIAKNTPKAPS-RLLS----------SR-ADDKTDVYPPHVMTQV---------DKLRA 145

ma|XP3810|MetarhiziumAcridum EWIAKDGTKAPG-GLLS----------SR-ADDGADIFSPHVMTQV---------DKLRA 149

mr|XP6681|MetarhiziumRobertsii EWIAQDGTKAPG-GLLS----------SR-ADDGVDTFSPHVMTQV---------DKLRA 149

ti|XP7451|TolypocladiumInflatum SSVGTPDP---N-FHDI--E-------ARANKTADDTFSTHAMTQV---------DKMRA 199

mr|XP4788|MetarhiziumRobertsii RVVGIPEA---G-KIS-----------SR-DGIRADTFSPHVMTQV---------DKLRA 137

pc|XP6498|PochoniaChlamydosporia RVVGTPAP---G-KVS-----------AR-GDKTADTFSPHIMTQV---------DKLRA 137

fg|XP6572|FusariumGraminearum HWTGTPGMEYTA-VQKE--E-------LQERDLSNDTYTPHVMTQI---------DKLRD 149

fx|XP7892|FusariumOxysporum HWTGTPGMEYKS-IQKR--G-------FEERDAANDTFSPHVMTQI---------DKLRA 151

fg|XP1472|FusariumGraminearum EWVAEPGMKAPL-SKR-------------AVNDTADTFSPHVMTQV---------DKLRK 150

fx|XP6097|FusariumOxysporum EWTATPGMKAPL-KKR-------------DLNDTADTFSPHVQVQV---------DKLRE 145

**MOTIFM3(LGGCFGxxC)**

**MOTIFM2 (AxxDxGxD)**

fx|XP2493|FusariumOxysporum GTTERVESDVVIALIDDG----VDKFEIGRP-D---QVLE-GKSFDFHD----ERVNPPY 853

fx|XP5158|FusariumOxysporum --TKPSLKPVEVALIDDG----VDFMHPDLN-DTRDRTFL-GKSFDYRDERPTPRVPPYW 787

mr|XP0588|MetarhiziumRobertsii KSADPALAPVKVALIDDG----ADITHPDLK-G---MKFP-GKSFHHYREGSSWRVSPFW 705

mr|XP5804|MetarhiziumRobertsii QGKDKQVAPVKIAIIDDG----IDATLHDLQ-S---KIAG-GATFCPYP-HSSELVNSYF 798

fx|XP5085|FusariumOxysporum F--VKPQNGIKVALIDDG----ADPYVESLR-G---KIWG-GETFSRGF-PHENGPSPYY 744

tr|XP7699|TrichodermaReesei D--ESIEEPIKVALIDDG----VDV--KDLE-F---NFIG-GRTFCTRD-EEHNLNDPYY 284

ti|XP9890|TolypocladiumInflatum AGV--RGRGVKVAIVDTG----VDYTHQAGT-I---A--------------------MSW 173

ma|XP1004|MetarhiziumAcridum AGM--RGKGVKVAVVDTG----IDYSHPALG-G---CFGP-GCKVAGGY----DLVGPEW 139

fg|XP6332|FusariumGraminearum AGI--RGKGATVAIVDTG----IDYTHKALG-G---CFGP-GCKVKGGY----DLVGADC 140

fx|XP5981|FusariumOxysporum ------------------------------------------------------------ 0

fx|XP5977|FusariumOxysporum AGI--RGKGSTVAIIDTG----IDYTHKALG-G---CFGP-GCKVKGGY----DLVGADW 188

fx|XP5978|FusariumOxysporum AGI--RGKGSTVAIIDTG----IDYTHKALG-G---CFGP-GCKVKGGY----DLVGADW 188

fx|XP5979|FusariumOxysporum AGI--RGKGSTVAIIDTG----IDYTHKALG-G---CFGP-GCKVKGGY----DLVGADW 188

fx|XP5980|FusariumOxysporum AGI--RGKGSTVAIIDTG----IDYTHKALG-G---CFGP-GCKVKGGY----DLVGADW 188

bb|XP3131|BeauveriaBassiana EGF--TGSGIRVAVIDTG----IDYTHPALG-G---CFGK-GCRVALGE----NFSQ-DG 215

ma|XP6042|MetarhiziumAcridum GTYYSVTLRLVAAVVDTG----IDYTNPALG-G---CFGK-GCRVAFGD----NFSK-DG 84

mr|XP4688|MetarhiziumRobertsii AGF--TGSGIRIAVVDSG----VDYTHPALG-G---CFGE-GCRVALGG----NFAK-DG 186

bb|XP2612|BeauveriaBassiana AGY--TGQGIRIAVVDSG----LNYTHSALG-G---CFGK-DCRVVLGD----NFSK-DG 184

bb|XP9611|BeauveriaBassiana AGF--TGSGIKIGIVDTG----LNYTHPAFG-G---CFGGDNCRVVAGD----NFSK-DG 188

cm|XP0114|CordycepsMilitaris EGY--SGKGIKIGIVDTG----VNYNIKALG-G---CFGP-NCRVAFGG----VFDG-KT 203

mr|XP1062|MetarhiziumRobertsii KGF--SGKGIKIAVVDTG----IDYTHPALG-G---CFGK-GCRVAFGD----NFSK-DG 193

bb|XP7963|BeauveriaBassiana EGL--SGNGIKIALVDTG----VDYTHPALG-G---CFGQ-HCRVVKGD----NLAK-DG 201

mr|XP5629|MetarhiziumRobertsii EGF--YGTNITIAVVDTGTGRQVNYTHPALG-G---CFGR-GCRVARGA----NFVRNEG 190

ma|XP9312|MetarhiziumAcridum KGI--TGKGVKIALLDSG----IDYEHPALG-G---CFGP-GCLVSFGA----DLLKNEP 212

bb|XP2014|BeauveriaBassiana KGI--TGKGIKIGMIDTG----VDYEHPALG-G---CFGP-GCLISFGA----DLVNNEP 204

ma|XP2468|MetarhiziumAcridum KGI--TGKGIKIAMIDTG----VDYEHPALG-G---CFGP-GCLFSFGA----DLVNNEP 206

mr|XP2804|MetarhiziumRobertsii KGI--TGKGIKIGMIDTG----VDYNHPALG-G---CFGP-GCLFSFGA----DLVNNDP 205

pl|XP0199|PurpureocilliumLilacinum LGI--TGNGVKIAIVDSG----VDYTHKSLG-G---CFGE-GCVISFGH----DFVGDDF 774

pl|XP9199|PurpureocilliumLilacinum KGI--TGKGVKVAILDTG----VSDFIV--------YSGS-WCPISFGY----DLVGDRY 156

pl|XP5939|PurpureocilliumLilacinum RGI--KGKGIKIAIADTG----VEYTHPALG-G---CFGP-GCHISFGT----DLVGDDY 192

pl|XP4570|PurpureocilliumLilacinum EGF--TGKGVKVAVIDTG----VDYTHEALG-G---CFGE-GCLVSFGT----DLVGDDY 257

pl|XP3158|PurpureocilliumLilacinum KGI--TGKGIKLAIVDTG----IDYTLKALG-G---CFGD-GCRVSFGY----DLVGDDY 191

pl|XP3207|PurpureocilliumLilacinum KGI--TGKGIKLAVIDSG----VDWKHPALG-G---CFGE-GCRISFGY----DLVGDNY 192

pl|XP5613|PurpureocilliumLilacinum KGI--TGKGIKLAIVDTG----VDYTQPSLG-G---CFGE-GCRVSFGY----DLVGDHY 193

pl|XP4279|PurpureocilliumLilacinum EGI--TGKGIKIAVIDTG----IDYTHPALG-G---CFGK-GCLVSFGR----DLVGDEY 194

pc|XP8386|PochoniaChlamydosporia KGF--NGTGVNVAIIDTG----VDYKHPSLG-G---CFGK-GCLVANGY----DFVGNNF 183

ma|XP1751|MetarhiziumAcridum KGY--TGKGVHVAVIDTG----IDYKHPSLG-G---CFGK-GCLVTKGF----DFVGDKF 184

mr|XP6614|MetarhiziumRobertsii KGY--TGKGVHVAVIDTG----IDYKHPSLG-G---CFGK-GCLVTKGF----DLVGDKF 184

pl|XP9153|PurpureocilliumLilacinum RGI--TGKGVKVAVIDTG----VDYTHPALG-G---CFGE-GCLVARGA----DFVGDAY 199

ti|XP3215|TolypocladiumInflatum KGI--TGKGIKVGVIDTG----IDYSHPALGNG---CFGS-GCLVARGY----DFVGDAY 186

bb|XP2472|BeauveriaBassiana KGY--KGRGIKVAVVDTG----IDYKHPALG-R---CFGE-GCLVGYGY----DLVGDAY 198

cm|XP74562|CordycepsMilitaris KGY--KGRGIKIAVIDTG----IDYKHPALG-R---CFGE-GCLVSYGY----DLVGDAY 197

ti|XP2193|TolypocladiumInflatum KGF--TGRGIKIAVIDTG----IDYKHPALG-G---CFGK-GCLVAFGT----DLVGDAY 159

pc|XP3398|PochoniaChlamydosporia KGI--TGKGVKIAVVDTG----IDYKHPALG-G---CFGK-GCLVAFGT----DLVGDAY 190

ma|XP3810|MetarhiziumAcridum KGV--TGKGVKIAVVDTG----IDYKHPALG-G---CFGH-GCLVAFGT----DLVGDAY 194

mr|XP6681|MetarhiziumRobertsii KGI--TGKGVKIAVVDTG----IDYKHPALG-G---CFGK-GCLVAFGT----DLVGDAY 194

ti|XP7451|TolypocladiumInflatum RGI--TGKGVKIAVIDTG----IDYTHPALG-G---CFGQ-GCLVSFGT----DLVGDAY 244

mr|XP4788|MetarhiziumRobertsii RGV--TGKGIKVAIIDTG----IDYTHPALGNN---CFGK-GCLVSFGT----DLVGDAY 183

pc|XP6498|PochoniaChlamydosporia KGI--TGKGVRVAIIDTG----IDYTHPALGNN---CFGK-GCLVSFGT----DLVGDDY 183

fg|XP6572|FusariumGraminearum EGV--TGKGLKVALVDSG----IDYKHPALG-G---CFGP-KCLVSFGT----DLVGDDY 194

fx|XP7892|FusariumOxysporum EGV--TGKGLKVALVDSG----IDYKHPALG-G---CFGK-NCLVSFGT----DLVGDAY 196

fg|XP1472|FusariumGraminearum KGI--TGHGIKVAVIDTG----IDYKHPALG-G---CFGP-DCLVSFGT----DLVGDDY 195

fx|XP6097|FusariumOxysporum KGI--TGHGIKVAVVDTG----IDYKHPALG-G---CFGP-NCLVSFGT----DLVGDKY 190

**MOTIFM4 (GHGxxVAG)**

fx|XP2493|FusariumOxysporum ----------------------------------LSAQGHGTTMASMILR---------- 869

fx|XP5158|FusariumOxysporum ----------------------------------SSPSGHGTLMARLIHK---------- 803

mr|XP0588|MetarhiziumRobertsii ----------------------------------DSSSGHGTLMARLIHR---------- 721

mr|XP5804|MetarhiziumRobertsii ----------------------------------VPRGKHGTLMAQLICD---------- 814

fx|XP5085|FusariumOxysporum ----------------------------------RSTKGHGTVMADMICR---------- 760

tr|XP7699|TrichodermaReesei ----------------------------------VSSTGHGTIMARQIQS---------- 300

ti|XP9890|TolypocladiumInflatum C------YP-----------------KYLTALPL-T------------------------ 185

ma|XP1004|MetarhiziumAcridum -----------------DSRSERTRPKKPDNNPM-DYKGHGTHVAGIIAGEN--EWLVLL 179

fg|XP6332|FusariumGraminearum GMFNVVCVTQQLTVYLGETHNEKKHPKRPDNDPM-DYQGHGTHVAGIIAAEN--EWL--- 194

fx|XP5981|FusariumOxysporum ---------------------------------M-DYQGHGTHVAGIIAAKN--EWL--- 21

fx|XP5977|FusariumOxysporum -----------------ETHNERKHPKQPDNDPM-DYQGHGTHVAGIIAAKN--EWL--- 225

fx|XP5978|FusariumOxysporum -----------------ETHNERKHPKQPDNDPM-DYQGHGTHVAGIIAAKN--EWL--- 225

fx|XP5979|FusariumOxysporum -----------------ETHNERKHPKQPDNDPM-DYQGHGTHVAGIIAAKN--EWL--- 225

fx|XP5980|FusariumOxysporum -----------------ETHNERKHPKQPDNDPM-DYQGHGTHVAGIIAAKN--EWL--- 225

bb|XP3131|BeauveriaBassiana D----------------------------KNDPM-DCQGHGTRVASILAE---------- 236

ma|XP6042|MetarhiziumAcridum K----------------------------DDDPM-DCHGHGTAVAGIVAGGD----S--- 108

mr|XP4688|MetarhiziumRobertsii K----------------------------DNDPM-DCNGHGTAVAGIVAGND----A--- 210

bb|XP2612|BeauveriaBassiana K----------------------------DKDPM-DCYGHGTTVAGILAGND----A--- 208

bb|XP9611|BeauveriaBassiana N----------------------------KDDPM-DCHGHGTAVAGVLAGND----A--- 212

cm|XP0114|CordycepsMilitaris S----------------------------KSDPM-DLHGHGTIVASILAGYS--KED--- 229

mr|XP1062|MetarhiziumRobertsii E----------------------------KGDPM-DCYGHGTQVAGVLAGYS--RDQ--- 219

bb|XP7963|BeauveriaBassiana K----------------------------KGDPM-DCVGHGTAVAGILAGYD--EKG--- 227

mr|XP5629|MetarhiziumRobertsii N----------------------------HDDPM-DHHGHGTAVAGVLAGND--PQR--- 216

ma|XP9312|MetarhiziumAcridum T-------------------------------PK-DCNGHGTQVAGIIAAKP--NPL--- 235

bb|XP2014|BeauveriaBassiana T-------------------------------PK-DCNGHGTNAVGIIAAQP--NEM--- 227

ma|XP2468|MetarhiziumAcridum T-------------------------------PK-DCNGHGTNAAGIIGARP--NPM--- 229

mr|XP2804|MetarhiziumRobertsii T-------------------------------PM-DCNGHGTNAAGIIGTRP--NAM--- 228

pl|XP0199|PurpureocilliumLilacinum D---------------------GYNTPHPKSDPM-DCFGHGTHVAGIIAAQPGTNRL--- 809

pl|XP9199|PurpureocilliumLilacinum D----------------PSSLDPTRRPAPDKDSM-DCHGHGTHVAGIIAARD--NPW--- 194

pl|XP5939|PurpureocilliumLilacinum T---------------------GYNDPVPDNYPN-DCNGHGTHVTGIIGALP--NPM--- 225

pl|XP4570|PurpureocilliumLilacinum H---------------------GSFSSIKPDNDPKDCNGHGTHVSGIIAAQK--NSL--- 291

pl|XP3158|PurpureocilliumLilacinum T---------------------GANVPVPDKDPM-DCAGHGTHVAGIVAAID--EDY--- 224

pl|XP3207|PurpureocilliumLilacinum D---------------------GYNMPEPDPDPRSTCNGHGTHITGIVAAKD--EAL--- 226

pl|XP5613|PurpureocilliumLilacinum T---------------------GSNVPVPDPDPL-DCAGHGTHVTGIVAAKD--EWL--- 226

pl|XP4279|PurpureocilliumLilacinum A----------------G----PGDEPAPDDDPR-DCEGHGTHVAGIIAARP--NPV--- 228

pc|XP8386|PochoniaChlamydosporia T---------------------GANTPVPSNDPM-DCTGHGTHVAGIVAAND--KKL--- 216

ma|XP1751|MetarhiziumAcridum D---------------------GKNARIPDDDPM-DCQGHGSHVAGIIAAAD--KKF--- 217

mr|XP6614|MetarhiziumRobertsii D---------------------GKNAPIPDDDPM-DCQGHGSHVAGIIAATD--EKF--- 217

pl|XP9153|PurpureocilliumLilacinum D---------------------GSNDPAPDDDPM-DCKGHGTHVAGIVAAQSTGNLF--- 234

ti|XP3215|TolypocladiumInflatum D---------------------GTNTPVEDNDPM-DCAGHGSHVAGIIAAQS--NKF--- 219

bb|XP2472|BeauveriaBassiana G----------------SN---GNYRPVPDNDPM-DCGGHGSHVAGIIAAQP--NEY--- 233

cm|XP74562|CordycepsMilitaris G-----------------N---GNYRPVPDNDPM-DCGGHGSHVAGIIAAQP--NEF--- 231

ti|XP2193|TolypocladiumInflatum D---------------------GGNTPHPDDDPM-DCAGHGSHVAGIVAAQP--NKF--- 192

pc|XP3398|PochoniaChlamydosporia T---------------------GFNTPQPDADPM-DCGGHGSHVAGIVAAQP--NTY--- 223

ma|XP3810|MetarhiziumAcridum D---------------------GSNTPHPDPDPM-DCGGHGSHVAGIVAAQP--NTF--- 227

mr|XP6681|MetarhiziumRobertsii D---------------------GSNTPHPDPDPM-DCGGHGSHVAGIVAAQP--NTY--- 227

ti|XP7451|TolypocladiumInflatum T---------------------GSNAPVPDNDPM-DCNGHGSHVAGIIAAQP--NRY--- 277

mr|XP4788|MetarhiziumRobertsii T---------------------GYNTPVPDNDPM-DCNGHGSHVAGIVAAQP--NPH--- 216

pc|XP6498|PochoniaChlamydosporia T---------------------GYNTPVPDNDPM-DCQGHGSHVAGIVAAQP--NVH--- 216

fg|XP6572|FusariumGraminearum D---------------------GFSQARPDNDPM-DCAGHGTHVAGILAAQK--NSM--- 227

fx|XP7892|FusariumOxysporum D---------------------GSNHAKPDNDPM-DCAGHGTHVAGILAAQK--NTM--- 229

fg|XP1472|FusariumGraminearum D---------------------GFNAVHPDDDPM-DCAGHGTHVAGIVAAQT--NPF--- 228

fx|XP6097|FusariumOxysporum D---------------------GFNAVYPDDDPM-DCQGHGSHVAGIVAAQE--NEY--- 223

**MOTIFM5 (GTSxxxP)**

fx|XP2493|FusariumOxysporum SGEGSKSGKNLTGSSVATALGAGLAAMIIYCVKASILSVKTANQ-NKAAI-----H-PIP 1064

fx|XP5158|FusariumOxysporum TQL--EKFEAHSGSSVANGLATGLAALVIECVRLGVIYTRELKR-LEPKVAFDYYHSSID 999

mr|XP0588|MetarhiziumRobertsii KQF--GRFAPHSGSSVATALAAGLAALIVECVRLGVLYTGETGP-LDETV-------TIG 914

mr|XP5804|MetarhiziumRobertsii ------SVVYDSGSSIATAAASGLAGLLIYSARLIYS-GTNEAR-N-----YPFHTQTAM 999

fx|XP5085|FusariumOxysporum ALP--DDFEERTGSSVATALADGLAALILHCVNLAVVHGKEHPS-T-----TAV-----S 954

tr|XP7699|TrichodermaReesei --D--TSSREVTGSSVATALAAGLAALVLYCVQVRLYLATDQEK-Q-----KA------- 482

ti|XP9890|TolypocladiumInflatum -----QQFKAQDGTSMAAPYIAGVAALYIGEHGGREVHGPGFARYLAKRI--------IS 370

ma|XP1004|MetarhiziumAcridum -----QGFETDSGSSMAAPYIAGIAALYIAHHGGRELHGPSFAKKLAQRI--------VA 403

fg|XP6332|FusariumGraminearum -----QSYEELSGTSMSAPYIAGIAALFIGKYGGRAFNGAGVAKMLRDRI--------AS 392

fx|XP5981|FusariumOxysporum -----QSYEELSGTSMSAPYIAGIAALFVGQYGGRAFNGAGFAKMLRDRI--------AS 219

fx|XP5977|FusariumOxysporum -----QSYEELSGTSMSAPYIAGIAALFVGQYGGRAFNGAGFAKMLRDRI--------AS 423

fx|XP5978|FusariumOxysporum -----QSYEELSGTSMSAPYIAGIAALFVGQYGGRAFNGAGFAKMLRDRI--------AS 423

fx|XP5979|FusariumOxysporum -----QSYEELSGTSMSAPYIAGIAALFVGQYGGRAFNGAGFAKMLRDRI--------AS 423

fx|XP5980|FusariumOxysporum -----QSYEELSGTSMSAPYIAGIAALFVGQYGGRAFNGAGFAKMLRDRI--------AS 423

bb|XP3131|BeauveriaBassiana -----GGYKEVSGTSYSGPFVGGIMALMAEVR-------GSFDPVLLNSL--------LT 418

ma|XP6042|MetarhiziumAcridum -----GGYEDISGTSFAGPLVAGILALAAEVR-------GPFDPVLLNSL--------LM 298

mr|XP4688|MetarhiziumRobertsii -----GGYEDITGTSFAGPLVAGILALVAEVR-------GTFDPVLLNSL--------LM 400

bb|XP2612|BeauveriaBassiana -----GGYGPVSGTSYAGPLVGGILALIAEVR-------GSFDPVFLNSL--------LM 398

bb|XP9611|BeauveriaBassiana -----GEYENVSGTSFSGPLVGGIMALMAEVR-------GSFDPALLNSL--------LM 402

cm|XP0114|CordycepsMilitaris -----GSYGSDSGTSFAAPLVAGIIALIAEAR-------GTFDPALIESL--------LM 408

mr|XP1062|MetarhiziumRobertsii -----GRYTYTSGTSYATPLVGGIVALVAEAR------GGNFNGVLINKL--------FM 400

bb|XP7963|BeauveriaBassiana -----GGYDKCSGTSYSGPQVAGIVALIAERR-------QNFDPALLNSL--------LM 424

mr|XP5629|MetarhiziumRobertsii -----GSYGNCSGTSFAGPQVAGMVALIAERR-------GDFDPGHLMSL--------LM 408

ma|XP9312|MetarhiziumAcridum ----ASGYGIRRGTSSSAPLIAGIVALIIEARRS-KTAGS-LGPVDIQNL--------LV 591

bb|XP2014|BeauveriaBassiana ----PSGYTVTRGTSFSGPLIAAIVALIGEAR------GS-LDPATVESL--------LV 578

ma|XP2468|MetarhiziumAcridum ----ISGYEITQGTSFSGPLIAAIVALIGEAR------GS-LDAATIESL--------LV 580

mr|XP2804|MetarhiziumRobertsii ----PSGYKITRGTSFSGPLISAIVALIGEAR------GS-LDPATVESL--------LV 579

pl|XP0199|PurpureocilliumLilacinum -----DRYAILDGTSMSCPLVAAAIALVAQVR------GT-SDNVLMRNL--------LS 1132

pl|XP9199|PurpureocilliumLilacinum ----KGTIEVMSGTSQATPLMAAIIALIFEVR------GK-LDPRSLTNL--------LA 522

pl|XP5939|PurpureocilliumLilacinum -----GTYVPQSGTSMATPFLAAAIALIAQVR------GT-RDPKTLNQL--------VS 566

pl|XP4570|PurpureocilliumLilacinum -----NDYANANGTSMSTPMVASILALIAQVR------GT-FDPTTLRNL--------VS 631

pl|XP3158|PurpureocilliumLilacinum -----G-YNIASGTSMATPLAAAIMALILQVR------GP-TAPKVLENL--------VS 564

pl|XP3207|PurpureocilliumLilacinum -----GEYYNTQGTSMSTPLTGAIMALILQVR------GP-TTPRSLNNL--------VS 566

pl|XP5613|PurpureocilliumLilacinum -----G-YSAASGTSMATPITAAILALILQVR------GP-TTPRMLDNL--------VS 567

pl|XP4279|PurpureocilliumLilacinum -----NTYAIRSGTSMATPLMAGIMALIAESR------GT-LDPALMMKL--------LS 572

pc|XP8386|PochoniaChlamydosporia ----KGSYAVASGTSMAAPLTAAMYALLMQIR------GR-NSPAMLQNL--------LS 557

ma|XP1751|MetarhiziumAcridum ----KGSYAVLSGTSMACPLTAAIYALLIEVR------GT-RDPMLLQNL--------LS 558

mr|XP6614|MetarhiziumRobertsii ----KGSYAVISGTSMACPLTAAIYALLVEVR------GT-RDPVLLQKL--------LS 558

pl|XP9153|PurpureocilliumLilacinum ----KGSYAVLSGTSMSCPLAAAIYALVGQVR------GR-IDPEEMENL--------LS 573

ti|XP3215|TolypocladiumInflatum ----KGSYAVMDGTSMSCPLTAAIYALIGQVR------GK-MDPSLFESL--------LS 557

bb|XP2472|BeauveriaBassiana ----KGSWAVLSGTSMATPFVAASVALLLEVR------GKKLPPATITNL--------LS 573

cm|XP74562|CordycepsMilitaris ----KGSYAVLSGTSMATPFIAGSVALLLEVR------GKKLAPATITNL--------LS 571

ti|XP2193|TolypocladiumInflatum ----LGGYAVLSGTSMACPIAAGIVALVAEVR------GT-FDPATIDSL--------LS 531

pc|XP3398|PochoniaChlamydosporia ----LGSYAVLSGTSMSCPQTAGILALIHEVR------GT-YDPELIQNL--------LS 564

ma|XP3810|MetarhiziumAcridum ----LGSYAVLSGTSMSCPQTAGIIALIHEVR------GT-YDPELIQNL--------LS 569

mr|XP6681|MetarhiziumRobertsii ----LGSYAVLSGTSMSCPQTAGIIALIHQVR------GT-YDPELIQNL--------LS 569

ti|XP7451|TolypocladiumInflatum ----KGGYAVLSGTSMSCPITAGIIALISEVR------GT-LDPELITNL--------LS 616

mr|XP4788|MetarhiziumRobertsii ----KGSYAVMSGTSMSCPITAAIITLVSEVR------GT-RDPRLINNL--------LS 555

pc|XP6498|PochoniaChlamydosporia ----KGSYAVLSGTSMSCPITAAIIALVSQVR------GT-RNPELINNL--------LS 555

fg|XP6572|FusariumGraminearum ----MGSYAVLSGTSMASPLVAGIVALIAEVR------GT-RDPALIENL--------LS 566

fx|XP7892|FusariumOxysporum ----MGSYAVLSGTSMASPLVAGIVALIAEAR------GT-RDPALIENL--------LS 568

fg|XP1472|FusariumGraminearum ----KGGYAVLSGTSMACPLVAATIALIAEVR------GT-LDPETIENL--------LA 568

fx|XP6097|FusariumOxysporum ----KGGYAVLSGTSMACPLVAGVIALIAEVR------GT-LDPEVLENL--------LS 564

*:* : . :
