# Supplementary material for: Phylogenetic analyses reveal molecular signatures associated with functional divergence among Subtilisin like Serine Proteases are linked to lifestyle transitions in Hypocreales
Source: BMC Evol Biol. 2016 Oct 19;16:220. doi: 10.1186/s12862-016-0793-y (PMC5069783; doi:10.1186/s12862-016-0793-y)
Supplement: Additional file 6: Figure S5. — Conserved Motifs identified by MEME in Proteinase K (S08.054). (DOCX 36 kb) [file 12862_2016_793_MOESM6_ESM.docx]

**FigureS5**.

**MOTIF M1 (GLxxxS)**

bb|XP7677|BeauveriaBassiana -------E-----TVTEDTCKPETEKQAPWGLARISHRETLGFGT------FNKYLYAEE 185

cm|XP6358|CordycepsMilitaris -------D-----TVTQDTCSPETEKQAPWGLARISHRQTLGFGT------FNKYLYAED 185

pc|XP3125|PochoniaChlamydosporia -------E----S--VQEDCNPETEKQAPWGLARVSHRKGLSFGT------YNKYLYAAE 182

ma|XP0725|MetarhiziumAcridum -------E----S--AQEDCTPETERQAPWGLARVSHRKGLSFGT------YNKYLYAAD 185

mr|XP6449|MetarhiziumRobertsii -------E----S--VQEDCTPETEKQAPWGLARVSHRKGLSFGT------YNKYLYAAD 185

fg|XP0192|FusariumGraminearum -------D-----MVTEDKCDGETERSAPWGLARISHRNTLNFGS------FNKYLYSSD 183

fx|XP5893|FusariumOxysporum -------N-----MITEDKCDGETERQAPWGLARISHRNTLNFGT------FNKYLYSSD 182

pl|XP1616|PurpureocilliumLilacinum -------D-----AVTEDTCDGETEKQAPWGLARVSHRKTLNFGT------FNKYLYAAE 186

ti|XP5718|TolypocladiumInflatum ------------HLVTEDKCNGETEKQAPWGLARVSHRNTLNFGT------FNKYLYAAD 181

tr|XP6166|TrichodermaReesei -------D----SIIVEDSCNGETEKQAPWGLARISHRETLNFGS------FNKYLYTAD 185

ma|XP5931|MetarhiziumAcridum -------VEAVEPDPKATAPVVGPQDQVPSHPDQVFRRDT----A------FTPYINNPT 155

mr|XP4522|MetarhiziumRobertsii -------VDAVEANPNATAPVVGPQGPVPSHPDQVFRRYN----G------LTQYLNNPT 155

ma|XP4373|MetarhiziumAcridum ----------VNRAPRNSTGAIIEQKGVTWNLDRISHQKSSSE-S------ETKYVYDSR 155

mr|XP9378|MetarhiziumRobertsii ----------VNRALQNSTGAPIEQKSATWNLGRISNQYSTSE-G------ESKYVYDSR 155

bb|XP1899|BeauveriaBassiana --------------------GYITQGRSSWGPARISHRKP----N------SNQYNYDES 138

cm|XP5458|CordycepsMilitaris --------------------GFVSQGGAPWGLSRLSSRWA----G------SDSYVFDDA 179

bb|XP2589|BeauveriaBassiana --------------------APATQSPSTWGLARISHETR----G------ASNYLYDSS 140

cm|XP9564|CordycepsMilitaris --------------------SGISQNPSAWGLSRISHKDP----G------TSDYLYDSS 132

pl|XP3276|PurpureocilliumLilacinum --------------------GYIEQPNAPWGLVRISHRQR----V------GTSYVYDDS 134

pl|XP1155|PurpureocilliumLilacinum --------------------GFESQPDATWGLSRISHRQA----G------ASTYDFDSS 136

pl|XP4677|PurpureocilliumLilacinum --------------------AYTQQPGAPWGLGRISHRSK----G------STTYEYDTS 136

pl|XP9521|PurpureocilliumLilacinum --------------------EFVTQPNATWGISRMSHRKP----H------ATDYVYDSS 121

pl|XP0926|PurpureocilliumLilacinum --------------------EYVTQSKATWGISRLSHLKP----H------IRNYTYDST 137

ma|XP1723|MetarhiziumAcridum --------------------AKVSQSDAPGNLVRLSKNLIDCV---------SNYDYDES 121

mr|XP6087|MetarhiziumRobertsii --------------------TSAAQQNAPGNLVRLSHYQTENV---------QTYEYDSS 74

mr|XP3684|MetarhiziumRobertsii -------------TLAKRNRAPKTQTSAPWGLRSISHRLPGAIYEGFPPSRNSEYYYDTN 110

ma|XP0847|MetarhiziumAcridum -------------ML--AKRALKTQKSAPWGLRAVSHRRAGAFYEKFPPDPASKYYYDDR 107

mr|XP4016|MetarhiziumRobertsii -------------ML--VKRASKTQKGAPWGLRAISHRRAGAFYEKFPPDPASKYYYDDR 107

bb|XP2820|BeauveriaBassiana ------------------LGRELTQTGAPWGLRAISHRDAHARPW-QTSLRRFEYFYDET 57

bb|XP0462|BeauveriaBassiana ------------------LAQELTQTGAPWGLRAISHRKPHARPR-QTSLRRFEYFYDKM 57

ma|XP3188|MetarhiziumAcridum ------------------TAEVQTQHKAPWNLRTISHRSSPRFPM-VNFFRNFKYYYQSW 61

bb|XP3476|BeauveriaBassiana ------------------AAEPQIQLKAPWNLRTISHREKPRFAS-VNVLRNFKYYYQSW 61

bb|XP0639|BeauveriaBassiana ------------------AAEPQVQLKAPWNLRTISHREKPKYAI-VNILRNFKHYYQSW 62

ma|XP5324|MetarhiziumAcridum --------------------ELVVQQNAPPGLQRLSEAAPVGQQQ-----QKGAYVFDSS 192

mr|XP6435|MetarhiziumRobertsii --------------------GLVKQENAPAGLQRLSEAAPVGEQP-----N--------- 178

bb|XP8622|BeauveriaBassiana --------------------LTFAQTNAPPGLARLSHNRTENS--------PNTYVYDSS 173

cm|XP2839|CordycepsMilitaris --------------------VVISQINAPPGLARLSHA--TNG--------PSNYLFDES 167

pl|XP3880|PurpureocilliumLilacinum --------------------AAIMQTNAPPGLNRISHARA-GE--------Q-GYIFDES 161

pc|XP9521|PochoniaChlamydosporia --------------------TTIVQTNAPPGLVRISEKRV-GD--------QGVYKFNDK 165

ti|XP8977|TolypocladiumInflatum --------------------ETIMQTNAPPGLNRLSHARA-GT--------Q-GYVFDDS 162

tr|XP7158|TrichodermaReesei --------------------ALVAQTNAAPGLIRLSNKAV-GG--------Q-NYIFDNS 131

fg|XP9382|FusariumGraminearum --------------------AAIAQVNAPPGLNRLSHAQA-DQ--------E-NYIFDDS 189

fx|XP3638|FusariumOxysporum --------------------AAIAQVNAPPGLDRLSHAKV-NQ--------D-TYVFDDS 185

fx|XP3639|FusariumOxysporum --------------------AAIAQVNAPPGLDRLSHAKV-NQ--------D-TYVFDDS 185

fg|XP0525|FusariumGraminearum -----------------------VQRNAEWGLASISHRTTGS----------TDYLYDAS 133

fx|XP1712|FusariumOxysporum ---------------------TITQQRSTWGLGSISHRTPHF----------NNYIYDSS 139

fg|XP3315|FusariumGraminearum --------------TELTDRALTTQSGAPWGLASISRRTSGG----------STYTYDTT 152

fx|XP9167|FusariumOxysporum -------------EPELADRALTTQSGAPWGLGTISHRTSGS----------TSYIYDTT 155

fg|XP8464|FusariumGraminearum DEQPTNEEEDDDDYQSLEKRGEANQTPSTWGLGTISHRRKGY----------NNYYYHKS 190

fx|XP9240|FusariumOxysporum TSQEGEDTEDEDEDDQLEKRSEINQKTSTWGLGTISHRNKGF----------QNYLYHKQ 185

fg|XP0806|FusariumGraminearum ---------PEK--ESLSKRAETTQSSATWGLGTVSHRSKGS----------TSYIYDTN 158

fx|XP5679|FusariumOxysporum ---------EEEEAETLAKRALTTQSGATWGLGTVSHRSRGS----------TSYIYDTN 161

fg|XP1405|FusariumGraminearum ---------D----HNLQKRAEVTQKKSTWNLGTISHRKKGF----------KEYIYDNY 159

fx|XP4666|FusariumOxysporum ---------K----RSLEKRDEITQKKSTWGLGTISHRKKGY----------KEYIYDDY 160

ma|XP5016|MetarhiziumAcridum -----------------IPMALTTQQNPPWALSAISSRTPGP----------QPYQYDDS 143

mr|XP6566|MetarhiziumRobertsii -----------------MPLALTTQQNPPWALSAMSSRTPGP----------QPYRYDDS 144

cm|XP0589|CordycepsMilitaris -----------------EELAWVKQDNAEHGLASLSSRGPGQ----------TAFSYEDT 138

cm|XP8384|CordycepsMilitaris -----------------KELVLVRQDNSDYGLASISNRGPAA----------TLYRYDDT 181

**MOTIF M3 (HGTxVAG)**

**MOTIF M2(HxxFxGRA)**

: :

bb|XP7677|BeauveriaBassiana -GGEGVDAYVIDTGTNIAHVDFEGRAKWGYTAPDG------DADEDGNGHGTHCSGTIAG 238

cm|XP6358|CordycepsMilitaris -GGEGVDAYVIDTGTNTEHVDFEGRAQWGYTAPEG------DADEDGNGHGTHCSGTIAG 238

pc|XP3125|PochoniaChlamydosporia -GGEGVDAYVIDTGTNIEHVDFEGRAKWGKTIPTG------DADEDGNGHGTHCSGTIAG 235

ma|XP0725|MetarhiziumAcridum -GGEGVDAYVIDTGTNTEHVDFEGRAKWGKTIPAG------DADEDGNGHGTHCSGTIAG 238

mr|XP6449|MetarhiziumRobertsii -GGEGVDAYVIDTGTNTGHVDFEGRAKWGKTIPAG------DADEDGNGHGTHCSGTIAG 238

fg|XP0192|FusariumGraminearum -GGEGVDAYIVDTGTNIKHVDFEGRAHWGKTIPSG------DADEDGNGHGTHCSGTVAG 236

fx|XP5893|FusariumOxysporum -GGEGVDAYIVDTGTNVDHVDFEGRAHWGKTIPSG------DADEDGNGHGTHCSGTVAG 235

pl|XP1616|PurpureocilliumLilacinum -GGEGVDAYVIDTGCNTEHVDFEGRAKWGKTIPSG------DADEDGNGHGTHCSGTIAG 239

ti|XP5718|TolypocladiumInflatum -GGEGVDAYVIDTGCNTEHVDFEGRAKWGKTIPTG------DEDEDGNGHGTHCSGTIAG 234

tr|XP6166|TrichodermaReesei -GGEGVDAYVIDTGTNIEHVDFEGRAKWGKTIPAG------DEDEDGNGHGTHCSGTVAG 238

ma|XP5931|MetarhiziumAcridum -GGQGVCAYVVDTGVDASHPDFGGRASMVASLVDSH-----GYD--FVGHGTHVAGILGS 207

mr|XP4522|MetarhiziumRobertsii -GGEGVCAYVVDSGVDVTHPEFGGRAHMVESTVDPH-----GLD--LVGHGTHVAGILGS 207

ma|XP4373|MetarhiziumAcridum -AGEGTCAYVIDTGVDDTRPEFKGRALQIKSFVANE-----TGDNSRDGHGTHVAGIIGS 209

mr|XP9378|MetarhiziumRobertsii -AGEGTCTYVIDTGVDDTHPEFEGRALQIKSFVANS-----TVDDSTDGHGTHVAGIIGS 209

bb|XP1899|BeauveriaBassiana -AGEGTCSYIIDSGIDADHPEFEGRATDLGRWGPGP-----DHD--DCMHGTHVAGIIGS 190

cm|XP5458|CordycepsMilitaris -AGEGTCSYIIDSGIDAQHPEFEGRATFLGHFGPGP-----HHD--DCGHGTHVAGTLGS 231

bb|XP2589|BeauveriaBassiana -SGSGTCSYIVDSGIDASHSDFGGRVEQIKSFIGP------NDD--SCGHGTHVAGTIGS 191

cm|XP9564|CordycepsMilitaris -AGAGTCSYVVDSGIDASHSDFDGRAELIKTFFGP------NKD--NCGHGTHVAGIIGS 183

pl|XP3276|PurpureocilliumLilacinum -AGLGTCAYIIDTGVDVTHPEFGNRAQVLKSFVND------TTD--GNGHGTHLAGIIGS 185

pl|XP1155|PurpureocilliumLilacinum -AGEGTCSYVIDTGVEADHPEFEGRAELLKSFIDGQ-----NAD--GNGHGTHVSGTIGS 188

pl|XP4677|PurpureocilliumLilacinum -GGSGTCAYVIDTGVEASHPEFEGRASQIKSFISGQ-----NTD--GNGHGTHCAGTIGS 188

pl|XP9521|PurpureocilliumLilacinum -AGEGTCAFILDTGINPDLAEFEGRAKMLKSYVDGE-----PTD--GHGHGTHVAGTIGS 173

pl|XP0926|PurpureocilliumLilacinum -AGEGTCAFVLDTGVFAEHPDFEGRAKMLKSYVPGQ-----DTD--GHGHGTHVSGTIGS 189

ma|XP1723|MetarhiziumAcridum -GGEGITVYVLDGGIRLTHQEFKGRATFGARFSKS------TSEEDQDGHGTHVSGIIGG 174

mr|XP6087|MetarhiziumRobertsii -AGQGITVYVLDGGIRLTHEEFEGRATFGAGFASH------QGEGDSNGHGTHVAAIIGG 127

mr|XP3684|MetarhiziumRobertsii -SGSGTFAYILDDGIRETHKEFEGRAKNIYSIFPE-------KQAGDYVHGTAVAGIIGS 162

ma|XP0847|MetarhiziumAcridum -AGLGTYAYILDGGIRTTHGEFEGRAETVFTLYPG-------DEIDHRGHGTGVAGVLGS 159

mr|XP4016|MetarhiziumRobertsii -AGLDTYAYILDSGIRTTHEEFEGRAETVFTVYPG-------DEIDHRGHGTAVAGVLGS 159

bb|XP2820|BeauveriaBassiana -AGEGTFGYVIDGGVRISHKELEGRAENLWTADLSPYPRNEPNYDDKTGHGTHIAGTIAS 116

bb|XP0462|BeauveriaBassiana -AGEGTFGYVIDGGVRITHKELEGRAENLWTADY-SYPGNEPNYDDTSGHGTHVAGIIAS 115

ma|XP3188|MetarhiziumAcridum PSDKTYYAYVVDSGVRISHKEFEGRAENLWTALKTR--DGKDDFEDKSGHGTHVAGTIAA 119

bb|XP3476|BeauveriaBassiana TSGDTYYAYVIDSGVRISHKEFEGRAENLWTAYKTS--EGKNDFEDKNGHGTHVAGIVAA 119

bb|XP0639|BeauveriaBassiana TSSESYYAYIVDSGIRITHKEFEGRAENFWTAYQTS--DGKDDFTDSTGHGTHVAGIVAS 120

ma|XP5324|MetarhiziumAcridum -AGNTTTAYVVDSGCLTTHQDFEGRATTIANLVRG------EKATDANGHGTHVACTIGG 245

mr|XP6435|MetarhiziumRobertsii ---------KVDSGCRTTHRDFEGRATTIANFVKG------ERATDANGHGTHVACTIAG 223

bb|XP8622|BeauveriaBassiana -AGENITAYVVDTGIRTSHIEFEGRAVLAANFVNN-------VDTDENGHGSHVAGTIGG 225

cm|XP2839|CordycepsMilitaris -AGQGVTVYIVDTGILPSHVEFEGRARSAANFVNQ-------VDTDENGHGSHVAGTIGG 219

pl|XP3880|PurpureocilliumLilacinum -ACEGMTVYVVDTGVRVDHVEFEGRAVMGANFVNN-------VDTDENGHGSHVAGTIAG 213

pc|XP9521|PochoniaChlamydosporia -GGEGITAYVVDTGVRVTHTEFEGRATFAANFVTN------SANTDENGHGSHVAGTIAG 218

ti|XP8977|TolypocladiumInflatum -AGAGITAYVVDTGVRVTHADFEGRATFAANFVNN-------VNTDEQGHGSHVAGTIAG 214

tr|XP7158|TrichodermaReesei -AGSNITAYVVDTGIRITHSEFEGRATFGANFVND--------DTDENGHGSHVAGTIGG 182

fg|XP9382|FusariumGraminearum -AGEGITAYVVDTGIKVDHSEFEGRATFGGNFIDN-------V----NGHGSHVAGTIGG 237

fx|XP3638|FusariumOxysporum -AGEGITAYVVDTGIKIDHSEFEGRATFGANFINN-------VDDDENGHGSHVAGTIGG 237

fx|XP3639|FusariumOxysporum -AGEGITAYVVDTGIKIDHSEFEGRATFGANFINN-------VDDDENGHGSHVAGTIGG 237

fg|XP0525|FusariumGraminearum -AGNGMYAYLVDTGINYGHRDFQGRASPGYNAYPG------VPFVDVNGHGTHCAGTIAG 186

fx|XP1712|FusariumOxysporum -AGAGTYAYVVDTGINIGHDEFQGRAALGYNAYPG------AEFVDANGHGTHCAGTIAG 192

fg|XP3315|FusariumGraminearum -AGSGSYGYVVDSGINVNHRDFGGRASLGYNAAGG-------AHVDTLGHGTHVAGTIAS 204

fx|XP9167|FusariumOxysporum -AGQGSYAYVVDSGVQVSHTNFGGRASLGYNAVGG-------AHEDTLGHGTHVAGTIAG 207

fg|XP8464|FusariumGraminearum -SGSDSYAYLVDSGVRTTHKEFQGRAKNGWTAFRR-------DFTDRLGHGTHVAGILAG 242

fx|XP9240|FusariumOxysporum -SGSDSFAYLVDSGVRTTHKEFQGRAKNGWTAFRK-------DYVDRLGHGTHVAGTIAG 237

fg|XP0806|FusariumGraminearum -AGSGTYAYIVDTGIITSHNEFEGRAQAVYTAFSG-------QNADTNGHGTHVAGTIAG 210

fx|XP5679|FusariumOxysporum -AGTNTYAYVVDTGVRTTHNEFEGRAQAVYTAFSG-------DNADSVGHGTHVSGTIAG 213

fg|XP1405|FusariumGraminearum -AGTDMYAYVIDGGVRVSHNEFGGRAKAAWTNWKG-------DNKDTDGHGTHVAGVIAG 211

fx|XP4666|FusariumOxysporum -AGTDMYAYVIDGGVRVTHNEFGGRAKAAWTNWKG-------NNKDDDGHGTHVAGTIAG 212

ma|XP5016|MetarhiziumAcridum -AGKDTFAYVLDSGVNANHVEFGGRATIGYSGYSNNNPN--KGVADRSGHGTMVAGLIAS 200

mr|XP6566|MetarhiziumRobertsii -AGENTFAYVLDSGVHDKHVEFGGRAAPGWSAYEEDFPD--RPHGDTSGHGTMVAGIIAS 201

cm|XP0589|CordycepsMilitaris -AAKGLFAYVVDSGIQADHPDFGGRVTRGFSINGNY--------DNIRPHGTMVAGVLAS 189

cm|XP8384|CordycepsMilitaris -AGKGTFGYVVDSGIQSSHPEFEGRVTQGYSFDGNF--------DNPTPHGTMVAGVLAS 232

:* * :: *. **: : :..

bb|XP7677|BeauveriaBassiana KKYGVAKKANVYAVKVLRSN------GSGSMSDVI-----KGVDWAAKSHEEQVKKAKDG 287

cm|XP6358|CordycepsMilitaris KKYGVAKKANVYAVKVLRSN------GSGTMSDVI-----KGVDWAASSHAEQVKKAKDG 287

pc|XP3125|PochoniaChlamydosporia KKYGVAKKANVYAVKVLRSN------GSGSMADVV-----KGVEFAATSHAEQVKAAKDG 284

ma|XP0725|MetarhiziumAcridum KKYGVAKKANVYAVKVLRSN------GSGTMADVV-----KGVEFAATNHVEQVLLAKDG 287

mr|XP6449|MetarhiziumRobertsii KKYGVAKKANVYAVKVLRSN------GSGTMADVV-----KGVEFAATSHVEQVLRAKDG 287

fg|XP0192|FusariumGraminearum KKYGVAKKASVYAVKVLRSN------GSGSMSDVV-----KGVEFAATSHLEQKKKAKDG 285

fx|XP5893|FusariumOxysporum KKYGVAKKANVYAVKVLRSN------GSGSMSDVV-----KGVEFAATSHLEQKKKAKDG 284

pl|XP1616|PurpureocilliumLilacinum KKYGVAKKASIYAVKVLRSN------GSGSMSDVV-----RGVEYAAQSHLAQVKAAKDG 288

ti|XP5718|TolypocladiumInflatum KKYGVAKKANVYAVKVLRSN------GSGSMSDVV-----KGVEYAATSHIEQAKAAKDG 283

tr|XP6166|TrichodermaReesei KKYGVAKKAHVYAVKVLRSN------GSGTMSDVV-----KGVEYAALSHIEQVKKAKKG 287

ma|XP5931|MetarhiziumAcridum NTYGVAKRVTIYGVKALSERPD-----ASGISNLI-----AGLNYVAEDA---------P 248

mr|XP4522|MetarhiziumRobertsii NSYGVAKRVTIYGIKALSERPD-----ASGISNMI-----AGLDYVARDA---------P 248

ma|XP4373|MetarhiziumAcridum ---GVAKKTRIFGIKVLNSE------GSRETDALIAGYASIGLEYVPIDA---------A 251

mr|XP9378|MetarhiziumRobertsii ASYGVAKKTKIFGIKVLDSN------GNAEGDRLIAGYAPTGLEYVPVDA---------A 254

bb|XP1899|BeauveriaBassiana KTYGVAKKTQLFGLKVLSYSAQDDGGCLGDNSDII-----AAVNAVAKDA---------A 236

cm|XP5458|CordycepsMilitaris RTYGVAKRTRLYGIKALGFIPE-IKKCAGDTSDII-----KAVNAVAHDA---------P 276

bb|XP2589|BeauveriaBassiana NTYGVAKKTTLLGIKVIDYNNR-TRECEGPTIGIL-----RGLEYVAQDV---------A 236

cm|XP9564|CordycepsMilitaris TTYGVAKKTSLYGIKVLSYDPV-LKQCSGSNDGII-----HGLEYVARDA---------A 228

pl|XP3276|PurpureocilliumLilacinum KTYGVAKKTRIYGVKVLDNS------GSGTYSNII-----AGMDFVAKDW---------K 225

pl|XP1155|PurpureocilliumLilacinum KTYGVAKKTQIYGVKVLDDQ------GSGPYSGII-----AGMDFVAKDA---------K 228

pl|XP4677|PurpureocilliumLilacinum KTYGVAKKTKIYGVKVLDNS------GSGSYSGII-----SGMDFAVQDS---------K 228

pl|XP9521|PurpureocilliumLilacinum KTFGLAKKTKLYGVKVLNNA------GTGPWSDVI-----AGLELVGRYV---------N 213

pl|XP0926|PurpureocilliumLilacinum KSFGVAKKTKIYGVKVLDNN------GNGNNTSIM-----EAMDWVAKFV---------R 229

ma|XP1723|MetarhiziumAcridum VKFGVAKKVKLVAVKLDP-----------EASQMI-----QALEFVLADV---------K 209

mr|XP6087|MetarhiziumRobertsii AKYGVAKQVQIVSVKLQP-----------REPQLE-----KALDFVLKDV---------Q 162

mr|XP3684|MetarhiziumRobertsii KTYGVAKKTTLLSVKTLGTT------GA-DHSEVL-----KALLWTAEHI---------V 201

ma|XP0847|MetarhiziumAcridum KTYGVAKRAKLLSVKTLDEE------GGCTASAAL-----RALSWTAEHI---------L 199

mr|XP4016|MetarhiziumRobertsii KTYGVAKRAKLLSVKTLDDK------GSCAASAAL-----HALSWTAEHI---------L 199

bb|XP2820|BeauveriaBassiana KTYGVAKKAHIVSVKVQNRF------GKTTSSNLL-----SGIHKAVKDI---------I 156

bb|XP0462|BeauveriaBassiana KTYGVAKKARIIAVKVPDRS------GTMTSSNLL-----SAIHRSVKDI---------L 155

ma|XP3188|MetarhiziumAcridum KTYGVAKTARVVSVRVLDKE------DRAPTSTII-----KGLEQAISDI---------A 159

bb|XP3476|BeauveriaBassiana KTYGVAKTAKVIAVRVLDEK------GGGTTAAIL-----KGMEQAIADI---------A 159

bb|XP0639|BeauveriaBassiana KTYGVAKSAKVVAVKVTNDK------GLTSTATII-----KGLEQAIADI---------A 160

ma|XP5324|MetarhiziumAcridum KNFGVAKKATVKCVKVMNAN------GQGQNADII-----AGLQSVVNDV---------Q 285

mr|XP6435|MetarhiziumRobertsii AKFGVAKLATVKCVKVMNAE------GQGTNADII-----AGLQSVVEDV---------K 263

bb|XP8622|BeauveriaBassiana ATYGVAKKAQLVGVKVLDKN------GAGANSGVL-----DGLQFVMNDV---------L 265

cm|XP2839|CordycepsMilitaris ATYGVAKRCELLGVKVLDAS------GGGANSGVL-----KGLQFVMNDV---------A 259

pl|XP3880|PurpureocilliumLilacinum ATFGVCKKANIVGVKVLGAD------GAGQNQGIL-----QGMQFVLNDV---------K 253

pc|XP9521|PochoniaChlamydosporia ATFGVAKNAQIKAVKVLDAQ------GRGENSAIL-----KGMEFVINDV---------T 258

ti|XP8977|TolypocladiumInflatum ATFGVAKRANIMAVKVLDAQ------GRGQNSDIL-----NGMQFCINDV---------Q 254

tr|XP7158|TrichodermaReesei ATFGVAKNVELVAVKVLDAD------GSGSNSGVL-----NGMQFVVNDV---------Q 222

fg|XP9382|FusariumGraminearum ATFGVAKKVDLVAVKVLDAS------GGGSNSGVL-----QGMQFVVDDV---------K 277

fx|XP3638|FusariumOxysporum ATFGVAKKVDLVAVKVLDAS------GGGSNSGVL-----QGMQFVIDDA---------K 277

fx|XP3639|FusariumOxysporum ATFGVAKKVDLVAVKVLDAS------GGGSNSGVL-----QGMQFVIDDA---------K 277

fg|XP0525|FusariumGraminearum KVYGVAKRANLIAVKVFHSG-------SSTTAIVL-----DGYNWAVNNI---------T 225

fx|XP1712|FusariumOxysporum KEYGVAKRANLIAVKVFHTG-------SSRTDIVL-----DGYNWAVTNI---------T 231

fg|XP3315|FusariumGraminearum STYGVAKAANVISVKVFTGN-------SASTSTIL-----AGFNWAVNDI---------T 243

fx|XP9167|FusariumOxysporum TTYGVAKRANIISVKVFAGR-------EGSTSTIL-----AGFNWAVNDI---------T 246

fg|XP8464|FusariumGraminearum KTYGVAKKAKIISVKVFQGD-------SADLSVIM-----TGIEWAVNDI---------I 281

fx|XP9240|FusariumOxysporum KTFGVAKKAKVISVKVFQGD-------SADLSVIL-----NGIEWAVNDI---------I 276

fg|XP0806|FusariumGraminearum KTYGVAKKATIQAVKVFQGS-------SSSTSIIL-----AGFNWAANDI---------I 249

fx|XP5679|FusariumOxysporum KTYGVSKKATIQAVKVFQGS-------SSSTSIIL-----AGFNWAANDI---------I 252

fg|XP1405|FusariumGraminearum KTYGVAKKANILALKVFKGE-------ESDTSIVL-----DAFNWAVNDI---------I 250

fx|XP4666|FusariumOxysporum KTYGVAKKANILALKVFNGD-------ESDTSIVL-----DAFNWAVNDI---------I 251

ma|XP5016|MetarhiziumAcridum NTYGVAKKANIIAVQSQ-----------NSASALL-----DSMSWAVQDI---------Q 235

mr|XP6566|MetarhiziumRobertsii NTYGVAKKANIIAVQTD-----------HTVSGTL-----GGIAWAVRDI---------Q 236

cm|XP0589|CordycepsMilitaris KTYGVAKNAQIIDVRANEIG------N-SKAAWVL-----SGINWAAKDI---------I 228

cm|XP8384|CordycepsMilitaris KTYGVAKKAEIINVRISSAQ------QRVTESSIL-----GGINWAVNDI---------V 272

*:.* : :: .

bb|XP7677|BeauveriaBassiana K-RKGFKGSVANMSLG-GGK------------TTLL-DMAVDAA---------VAKGLHF 323

cm|XP6358|CordycepsMilitaris K-RKGFKGSVANMSLG-GGK------------TTLL-DQAVDAA---------VSKGLHF 323

pc|XP3125|PochoniaChlamydosporia K-RKGFKGSVANMSLG-GGK------------TQAL-DAAVNAA---------VKAGIHF 320

ma|XP0725|MetarhiziumAcridum K-RKGFKGSVANMSLG-GGK------------TQAL-DAAVNAA---------VKAGIHF 323

mr|XP6449|MetarhiziumRobertsii K-RKGFKGSVANMSLG-GGK------------TQAL-DAAVNAA---------VKAGIHF 323

fg|XP0192|FusariumGraminearum K-RKGFKGSVANMSLG-GGK------------TQAL-DAAVNAA---------VRTGIHF 321

fx|XP5893|FusariumOxysporum K-RKGFKGSVANMSLG-GGK------------TQAL-DAAVNAA---------VRTGIHF 320

pl|XP1616|PurpureocilliumLilacinum K-RKGFKGSVANMSLG-GGK------------TQAL-DAAVNAA---------VKSGIHF 324

ti|XP5718|TolypocladiumInflatum K-RKGFKGSVANMSLG-GGK------------TQAL-DAAVNAA---------VRSGVHF 319

tr|XP6166|TrichodermaReesei K-RKGFKGSVANMSLG-GGK------------TQAL-DAAVNAA---------VRAGVHF 323

ma|XP5931|MetarhiziumAcridum R-RSCPNGIVVNMSAGVPRI------------IPAL-NLAARKL---------VQRGFFV 285

mr|XP4522|MetarhiziumRobertsii H-RHCPNGIVVNLSAGIAER------------NDAL-NMAARGL---------VERGYFV 285

ma|XP4373|MetarhiziumAcridum N-RTCPNGVVVSFSINSGLY------------EKAI-SVAAGAL---------VKKGYFM 288

mr|XP9378|MetarhiziumRobertsii N-RTCPNGVVVNYSINSNGY------------AKSI-NVAAAEL---------AKKGYFV 291

bb|XP1899|BeauveriaBassiana E-RHCPNGVFVNMSLG-GGY------------SRAL-NDAVDNL---------AGRGVFV 272

cm|XP5458|CordycepsMilitaris G-RDCPKGVIVNMSLG-GGY------------SQAL-NDAVDNL---------SARGIFV 312

bb|XP2589|BeauveriaBassiana N-RNCPKGVVVNMSLG-GSF------------SQAS-NDAVAEL---------VSKGFFV 272

cm|XP9564|CordycepsMilitaris N-RSCPNGVVVNMSLG-GGF------------TQML-NDAVAAL---------VSRGYFV 264

pl|XP3276|PurpureocilliumLilacinum T-RNCPKGAMANLSLG-GSF------------SAAV-NNAAASL---------VKAGVFT 261

pl|XP1155|PurpureocilliumLilacinum T-RGCPKGAVANMSLE-GGY------------AASV-NNAAASL---------VQSGVFV 264

pl|XP4677|PurpureocilliumLilacinum T-RSCPKGVVANMSLG-GGK------------AQSV-NDGAAAM---------IRAGVFL 264

pl|XP9521|PurpureocilliumLilacinum C-TKCPKGVVVNMSLA-GKK------------IDTV-NQAAKSL---------VEAGAFL 249

pl|XP0926|PurpureocilliumLilacinum C-GQCPKGVYVNLSLR-GRF------------SVAF-NNAAAAL---------VKAGVFV 265

ma|XP1723|MetarhiziumAcridum K-KGIQGKAVISMSMHV-DG------------SEIV-DKKFKHL---------VDSGVVV 245

mr|XP6087|MetarhiziumRobertsii H-RNITGKAIISMSMSF-PA------------SDDI-DKMFRRL---------VNSGIVC 198

mr|XP3684|MetarhiziumRobertsii N-NTRQKSSVINLSFGV-EK------------SDAL-NKFIELLV--------GKYDIPV 238

ma|XP0847|MetarhiziumAcridum R-NGRQHSSVINLSFGV-KK------------VQSL-NTFIEALI--------SEADVPV 236

mr|XP4016|MetarhiziumRobertsii S-NGRQHSSVINLSFGI-PK------------LQAL-DTFIEALV--------SQVGIPV 236

bb|XP2820|BeauveriaBassiana D-KGRVGKAVINISLGG-GC------------QERAVSQAIQRA---------VTNGITI 193

bb|XP0462|BeauveriaBassiana D-KGRVGKAVINLSLGG-SC------------QQKSISQAITRA---------VANGITV 192

ma|XP3188|MetarhiziumAcridum K-KNRHNNAVINMSVGA-EC------------STAM-NTIIQRAYKRRDASGKGLASILV 204

bb|XP3476|BeauveriaBassiana R-KDRHNHAVINMSIGM-PC------------STAI-NTVIDRAYSRRDDSQRNPAGILF 204

bb|XP0639|BeauveriaBassiana K-KDRHNNAVINISLGV-EC------------SQAM-NTIIDRAYSRRDASYRNPAGILV 205

ma|XP5324|MetarhiziumAcridum K-NNLQGKAVVNLSLGG-GK------------SQAL-DAAMNNV---------FKAGIVP 321

mr|XP6435|MetarhiziumRobertsii K-TKPQ-AATMNMSLGG-GR------------SQAL-DTAINNV---------FKAGVLP 298

bb|XP8622|BeauveriaBassiana K-RGLGGKSVMNMSLGG-GR------------SEAV-NRAIQQL---------FAAGIVP 301

cm|XP2839|CordycepsMilitaris E-RNV-SKAVMNMSLGG-PR------------SRAI-NTAISQL---------AQAGVIP 294

pl|XP3880|PurpureocilliumLilacinum S-KGIQNKAVMNMSLGG-GF------------SQAL-NRAIAAL---------LDGGIVP 289

pc|XP9521|PochoniaChlamydosporia K-NNLKGKAVMNMSLGG-SK------------SQAM-NRAIQAV---------FNAGIVP 294

ti|XP8977|TolypocladiumInflatum Q-RGIQGKAVMNMSLGG-SF------------SQAM-NRAIQAV---------RNAGIVP 290

tr|XP7158|TrichodermaReesei A-KKRSGKAVMNMSLGG-SF------------STAV-NNAITAL---------TNAGIVP 258

fg|XP9382|FusariumGraminearum K-NNRAGKAVMNMSLGG-DK------------SEAI-NRAIEAL---------FKAGVVP 313

fx|XP3638|FusariumOxysporum K-KNRVGKAVMNMSLGG-DF------------SQAI-NRAIEAL---------FKAGIVP 313

fx|XP3639|FusariumOxysporum K-KNRVGKAVMNMSLGG-DF------------SQAI-NRAIEAL---------FKAGIVP 313

fg|XP0525|FusariumGraminearum NTPGRNQQSVISMSLGG-GK------------SDAF-NLAVEMA---------YRQNIHT 262

fx|XP1712|FusariumOxysporum NTPGRKEQAVISMSLGG-SR------------SDAF-NAAVQAA---------YSAGVHT 268

fg|XP3315|FusariumGraminearum S-KGRAGRSVINMSLGG-PS------------AQTW-TTAINAA---------YNSGVLS 279

fx|XP9167|FusariumOxysporum S-KSRAGRSVINLSLGG-PA------------SQTW-TSAINAA---------YNSGVLS 282

fg|XP8464|FusariumGraminearum K-KKRQEFSVINLSLGVNGV------------SGAL-NDIIKNA---------AKAGVII 318

fx|XP9240|FusariumOxysporum K-KNRQEFSVINLSLGIDGV------------SGAL-NDIIKNA---------AKSGVIF 313

fg|XP0806|FusariumGraminearum S-KGRTKTSVVNMSLGG-GY------------SASF-NNAVESA---------SSSGIIS 285

fx|XP5679|FusariumOxysporum S-KSRTARSVVNMSLGG-GY------------SASF-NNAVDSA---------SRSGIIS 288

fg|XP1405|FusariumGraminearum K-RDRTWRAVINMSLGG-EK------------SVAF-NRAVDNA---------SKKGVVT 286

fx|XP4666|FusariumOxysporum K-KDRTWRAVINMSLGG-EK------------STAF-NKAVDTA---------SKKGVVT 287

ma|XP5016|MetarhiziumAcridum K-QGRVGRAVINYSGGIQKFADSNP-FVTESPGISM-ARIMETA---------FNQGILC 283

mr|XP6566|MetarhiziumRobertsii S-QGRVGQAVINYSGGLETIPDSAG-YK-YQPGVAM-AQSMDIA---------FNEGILC 283

cm|XP0589|CordycepsMilitaris D-KGRTRQAVINVSLNAKASVAKVSDMFAPPDKSPV-NLAVEAA---------FKLGVLT 277

cm|XP8384|CordycepsMilitaris D-RGRVGKAVINVSQTGG---------ANPNGPSPI-NSAVEAA---------FRLGILT 312

**MOTIF M4 (DxxAPG)**

. *

bb|XP7677|BeauveriaBassiana AVAAGNDN--------ADACNYSPAAAKKAVTVGASTIDDSRA-YFSNYGKCTDIFAPGL 374

cm|XP6358|CordycepsMilitaris AVAAGNDN--------ADACNYSPAAAKKAVTVGASAIDDSRA-YFSNYGKCTDIFAPGL 374

pc|XP3125|PochoniaChlamydosporia AVAAGNDN--------ADACNYSPAAAELPVTVGASAFDDTRA-YFSNYGKCTDIFAPGL 371

ma|XP0725|MetarhiziumAcridum AVAAGNDN--------ADACNYSPAAAELPVTVGASAFDDSRA-YFSNYGKCTDIFAPGL 374

mr|XP6449|MetarhiziumRobertsii AVAAGNDN--------ADACNYSPAAAELPVTVGASAFDDSRA-YFSNYGKCTDIFAPGL 374

fg|XP0192|FusariumGraminearum AVAAGNDN--------ADACNYSPAAASEPVTVGASAIDDSRA-YFSNYGKCTDIFAPGL 372

fx|XP5893|FusariumOxysporum AVAAGNDN--------ADACNYSPAAASEPVTVGASALDDSRA-YFSNYGKCTDIFAPGL 371

pl|XP1616|PurpureocilliumLilacinum AVAAGNDN--------ADACNYSPAAATEPVTVGASALDDSRA-YFSNYGKCTDIFAPGL 375

ti|XP5718|TolypocladiumInflatum AVAAGNDN--------ADACNYSPAAASEPVTVGASALDDSRA-YFSNYGKCTDIFAPGL 370

tr|XP6166|TrichodermaReesei AVAAGNDN--------ADACNYSPAAATEPLTVGASALDDSRA-YFSNYGKCTDIFAPGL 374

ma|XP5931|MetarhiziumAcridum AVAAGNEG--------HDARVNSPASEPSICTVGGF-----GY-RDSNYGSVVDIQAPGA 331

mr|XP4522|MetarhiziumRobertsii AVAAGNER--------HDARLNSPASEPSICTVGDY-----RY-RDSNFGPAVDIQAPAV 331

ma|XP4373|MetarhiziumAcridum AASAGNI-----------VAHSSPASEATVCTVGSVDINNKPA-SDYGYGPGLDLLAPGV 336

mr|XP9378|MetarhiziumRobertsii AVAAGNKP--------RDVAQSSPASEATVCTVGSVDINNKPA-VDTGYGPGVDLMAPGV 342

bb|XP1899|BeauveriaBassiana AVAAGNKN--------KDASGVSPASAKNVCCVGGTDSSDHRY-VDSNYGANVDVAAPGV 323

cm|XP5458|CordycepsMilitaris AAASGNTN--------TDARGDSPASASRACVVGATDRWDRRF-EMSNFGPNIDINAPGV 363

bb|XP2589|BeauveriaBassiana AVAAGNGDEKKG---PMDAGDVSPASERSACTVGASDRNDRIA-HFSNYGSMIDIHAPGV 328

cm|XP9564|CordycepsMilitaris GVAAGNGDEHNN---PIDAGDVSPASEPSACTVGASDANDEVA-TFSNYGDVVDLHAPGV 320

pl|XP3276|PurpureocilliumLilacinum SVAAGGDN--------SDASRNSPASEPTVCTVGGTTRSDTRL-SSSNYGPIVDIFAPGE 312

pl|XP1155|PurpureocilliumLilacinum GVAAGNSA--------KDAGESSPASEPSVCTVGATDRNDNKS-SFSNYGDVLDIFGPGT 315

pl|XP4677|PurpureocilliumLilacinum AVAAGNDN--------ANAANYSPASEPTVCTVGATTSSDARS-SFSNYGSLVDIFAPGS 315

pl|XP9521|PurpureocilliumLilacinum GVAAGNFG--------DDAANWSPASEPSVCTVAASTADDTMP-YWSNFGELVDVFAPGA 300

pl|XP0926|PurpureocilliumLilacinum GVSAGNDG--------DNAGSYSPGAEPTVCTVGASDNDDNVL-LWSNYGEVVDVFAPGQ 316

ma|XP1723|MetarhiziumAcridum VMDAG---------------QFSPGRDPSVITVAAMDHRNDFHWSKSNYGPSVTIYAPGV 290

mr|XP6087|MetarhiziumRobertsii VVSAGNNNW--------DASKNSPSRDPGVITVAAMNHRSDSRWEESSYGPAVDLYAPGA 250

mr|XP3684|MetarhiziumRobertsii VTAAGNEGE--------DASTKTPGSAKGAINVGYINKQWGLA-PRSNWGPAVTILAPGV 289

ma|XP0847|MetarhiziumAcridum VTAAGNENE--------DASISTPGSAKGVINVGHMNKHWVLS-PKSNWGPSVTMLAPGV 287

mr|XP4016|MetarhiziumRobertsii VTAAGNENE--------DASLSTPGSAKGVINVGHMDKNWVMS-PNSNWGPAVTMLAPGV 287

bb|XP2820|BeauveriaBassiana VQASGNEGE--------DAKDYCLAPKAGAITVGNMKPDWTIH-NTSNFGSTVDIFGPGT 244

bb|XP0462|BeauveriaBassiana VQAAGNNGK--------DAKDHCLAPNARAITVGNMDQDWTAY-STSNYGSTVDIFGPGT 243

ma|XP3188|MetarhiziumAcridum VAASGNEGA--------DASTCSPASSNDALTVGAIDSSWNVV-KWSNYGRKVDILAPGD 255

bb|XP3476|BeauveriaBassiana IVCSGNEAI--------NARDCSPASARQAITVGAVNPKWNVV-SWSNFGSTVDILAPGI 255

bb|XP0639|BeauveriaBassiana VVASGNEAM--------NAKECSPASARQALTVGAIGPNWNVV-SWSNFGSSVDILAPGD 256

ma|XP5324|MetarhiziumAcridum VVAAGNENACANRVLQQDAANTSPASARNAITVGAVDANTDQKASFSNFGRDVDINAPGV 381

mr|XP6435|MetarhiziumRobertsii VQN---------------AKNVSPAAAPNAITVGAVDANTDQKAGFSNFGPSVDINAPGV 343

bb|XP8622|BeauveriaBassiana VVAAGNEN--------QDAANTSPASAKAAICVGAINAKNDVKASFSNKGEAVDIFAPGV 353

cm|XP2839|CordycepsMilitaris VVAAGNED--------QDASTTSPASAPDAITVGAIDVRNDAKASFSNFGSSVAIFAPGV 346

pl|XP3880|PurpureocilliumLilacinum VVAAGNEK--------QDARNTSPASAPQAITVGAIDATNDQFASFSNFGSVVDVNGPGV 341

pc|XP9521|PochoniaChlamydosporia VVAAGNEN--------QDTANTSPGSAPSAITVGAIDATTDSRATFSNFGQSVDIFAPGV 346

ti|XP8977|TolypocladiumInflatum VVAAGNEN--------QDTANTSPGSAPSAITVGAIDGKNDTRASFSNFGKAVDIFAPGV 342

tr|XP7158|TrichodermaReesei VVAAGNEN--------QDTANTSPGSAPQAITVGAIDATTDIRAGFSNFGTGVDIYAPGV 310

fg|XP9382|FusariumGraminearum VVAAGNEN--------RETALTSPGSAPNAITVGAIDATSDQRADFSNFGPEVDIYAPGV 365

fx|XP3638|FusariumOxysporum VVAAGNEN--------RETALTSPGSAPNAITVGAIDATTDERADFSNFGPEVDVYAPGV 365

fx|XP3639|FusariumOxysporum VVAAGNEN--------RETALTSPGSAPNAITVGAIDATTDERADFSNFGPEVDVYAPGV 365

fg|XP0525|FusariumGraminearum VVAAGNSNV--------NANDTSPASAQNATTVGAIDKNNNRA-SFSNFGPFVDIFAPGV 313

fx|XP1712|FusariumOxysporum VVAAGNDNA--------DAAKYSPASAPNATTIGAIDVDNKRA-SFSNYGELVDLFAPGV 319

fg|XP3315|FusariumGraminearum VVAAGNGDDLGR---PLPVSGQSPANVPNALTVAAIDSSWRTA-SFTNYGAGVDVFAPGV 335

fx|XP9167|FusariumOxysporum VVAAGNGDDAGR---PLPVSGQSPANAPNALTVAAIDSSWRPA-SFTNYGAGVDVFGPGV 338

fg|XP8464|FusariumGraminearum VVAAGNQGK--------SASLTSPSSAPQAITVGAIDNNWKIP-SWSNYGSSVDILAPGV 369

fx|XP9240|FusariumOxysporum VVAAGNQGT--------DAKTKSPASAPQAITVGAIDKNWRIP-SWSNYGSSVDMMAPGV 364

fg|XP0806|FusariumGraminearum AIAAGNDGA--------NAANTSPASAPSAITVGAIDSNWAIA-SYSNYGTVLDIFAPGT 336

fx|XP5679|FusariumOxysporum AIAAGNDGA--------NAANTSPASAASAITVGAIDSNWAIA-SYSNYGTVLDIFAPGS 339

fg|XP1405|FusariumGraminearum VVASGNDAI--------DAAKESPGSAATTITVGAIDENWAVA-DFSNYGKTVDIMAPGV 337

fx|XP4666|FusariumOxysporum VVASGNDAI--------DAAKESPGSASTAITVGAIDSNWAVA-DFSNWGKTVDILAPGV 338

ma|XP5016|MetarhiziumAcridum VIASGNQGVVVE---Q--SDAPYQGNSTSALVVGAVDEKWGHA-SFSNYGPSVDILAPGA 337

mr|XP6566|MetarhiziumRobertsii VIAAGNDGQVVE---Q--SSTPYQGNSTTALAVGAINQQWNFM-RLSNHGPSVDILAPGE 337

cm|XP0589|CordycepsMilitaris VAAAGNEN-TSD---P---VTVTPGSARNALTVGAIDRDWKFSGNFSNHGSMVDILAPGT 330

cm|XP8384|CordycepsMilitaris IAAAGNAFGTVE---S---ESITPGSAPNALTVGAIDKSWKFS-DFSNHGKMVDILAPGT 365

**MOTIF M5 (GTSxAxP)**

. :. * : .*.

bb|XP7677|BeauveriaBassiana NIQSTWIGSKY----------------AINTISGTSMASPHICGLLAYYLSLQPA---SD 415

cm|XP6358|CordycepsMilitaris NIQSTWIGSKY----------------AINTISGTSMASPHICGLLAYYLSLQPA---SD 415

pc|XP3125|PochoniaChlamydosporia NILSTWIGSKY----------------AVNTISGTSMASPHICGLLAYYLSLQPA---SD 412

ma|XP0725|MetarhiziumAcridum NILSTWIGSKT----------------AVNTISGTSMASPHICGLLAYYLSLQPA---GD 415

mr|XP6449|MetarhiziumRobertsii NILSTWIGSPT----------------AVNTISGTSMASPHICGLLAYYLSLQPA---GD 415

fg|XP0192|FusariumGraminearum NIVSTWIGTDK----------------AINTISGTSMASPHIAGLLAYYLSLQPA---ED 413

fx|XP5893|FusariumOxysporum NIQSTWIGSKY----------------AVNTISGTSMASPHIAGLLAYYLSLQPA---ED 412

pl|XP1616|PurpureocilliumLilacinum SIQSTWIGSKY----------------AVNTISGTSMASPHICGLLAYYLSLQPA---GD 416

ti|XP5718|TolypocladiumInflatum SIQSTWIGSKY----------------AVNTISGTSMASPHICGLLAYYLSLQPA---GD 411

tr|XP6166|TrichodermaReesei SIQSTWIGSKY----------------AVNTISGTSMASPHICGLLAYYLSLQPA---GD 415

ma|XP5931|MetarhiziumAcridum NILSTVPGGGA------------------VSHQSHGHTLTL------------------- 354

mr|XP4522|MetarhiziumRobertsii NVLSTVPGGRI------------------YRLTGTSMASPYIAGLAASIASAHHQ---RA 370

ma|XP4373|MetarhiziumAcridum DILSLQPDNRT------------------ALLSGTSQATPHVTGLAAYFASIFGK---SA 375

mr|XP9378|MetarhiziumRobertsii DIMSLQPDNRT------------------SLLSGTSMATPHVTGLAAYFASIYGK---SA 381

bb|XP1899|BeauveriaBassiana VVLSTFPNGRT------------------GYMTGTSMATPHVAGLAAYLAAKDGV---SG 362

cm|XP5458|CordycepsMilitaris DILSTMPNGQV------------------GTMTGTSMASPHIAGLAAYLAAKDGL---SG 402

bb|XP2589|BeauveriaBassiana DIVSLKNGGGT------------------ALGSGTSMATPHVAGLAAYFMGQGRA----- 365

cm|XP9564|CordycepsMilitaris SIVSLRAGGGT------------------ISMDGTSMATPHIVGLAAYFMGQGKS----- 357

pl|XP3276|PurpureocilliumLilacinum AITSTWTNGGTVSRDASPLGTLRSHMILQNTISGTSTSAAYITGLGCYLAGFEGN---PG 369

pl|XP1155|PurpureocilliumLilacinum DVMSTWIGGQI------------------KTISGTSMATPHIVGLAAYLAALEGK---TG 354

pl|XP4677|PurpureocilliumLilacinum NILSTWIGGTT------------------NTISGTSMATPHIVGLGAYLAGLEGF---PG 354

pl|XP9521|PurpureocilliumLilacinum SINSTWFDGTT------------------HLSSGTSMASPHVVGLAAYLGALEGI---KG 339

pl|XP0926|PurpureocilliumLilacinum DIQSTWIDGGW------------------NYLDGTSMSTPHVVGLAAYLGALEGI---SG 355

ma|XP1723|MetarhiziumAcridum GIESSYFRSDT----------------ATRYLNGTSQATPHVAGLAAYIMALEGI---TQ 331

mr|XP6087|MetarhiziumRobertsii DITSASRDSDS----------------ASVTLSGTSQAVPHVAGLAAYIMSLESI---TK 291

mr|XP3684|MetarhiziumRobertsii DVETTGSESDT----------------NAVLQSGSSYAAPYISGLVLNAISVHGV---KG 330

ma|XP0847|MetarhiziumAcridum DVECPSSGSDT----------------NVILQSGSSFAAPHVAGLVLNAISVHGI---KG 328

mr|XP4016|MetarhiziumRobertsii QVECPSSASDT----------------NVVLESGSSFAAPHVAGLVLNAISVHGI---KG 328

bb|XP2820|BeauveriaBassiana DILSLSSADDK----------------KLQLMTGTSMAAPHVAGVALTFMSASGS---PK 285

bb|XP0462|BeauveriaBassiana DILSLSNADDK----------------KSQLMTGTSMAAPHVAGVALTFMS--GS---PK 282

ma|XP3188|MetarhiziumAcridum GVTSLSSKSDY----------------GTETMSGTSMAAPHVAALALNAMAVFSK---LS 296

bb|XP3476|BeauveriaBassiana DIVSLSNKSDT----------------ETATKSGTSMATPHVAALALNAMAVFSK---IS 296

bb|XP0639|BeauveriaBassiana DITSLSSKSDT----------------GTITHSGTSMASPHVAALALNAMSVFSK---IS 297

ma|XP5324|MetarhiziumAcridum KVQSCGINSNT----------------DVSVKSGTSMASPHVAGLAAYLMTLENI---DS 422

mr|XP6435|MetarhiziumRobertsii DVQSCGIRSDS----------------DVSTKSGTSMASPHVAGLANYLMRLENV---SD 384

bb|XP8622|BeauveriaBassiana DILSVGIDSDT----------------ATNTLSGTSMASPHVAGLAAYFLGLESN-L-NQ 395

cm|XP2839|CordycepsMilitaris NILSVGIASNT----------------DSKVLSGTSMASPHVAGLAAYLMGLRGLDE-QQ 389

pl|XP3880|PurpureocilliumLilacinum KVLSVGIASTV----------------ATNVLSGTSMASPHVAGLTAYVMGLTGR----Q 381

pc|XP9521|PochoniaChlamydosporia KILSVGIGSDI----------------ETKVLSGTSMASPHVAGLAAYLMSFQSF---QT 387

ti|XP8977|TolypocladiumInflatum NVLSVGIRSDV----------------DTKVLSGTSMASPHVAGMAAYLMGLNLKNVNNP 386

tr|XP7158|TrichodermaReesei DVLSVGIKSDI----------------DTAVLSGTSMASPHVAGLAAYLMALEGV---SN 351

fg|XP9382|FusariumGraminearum DVLSVGIKSNT----------------DTATLSGTSMASPHVAGLAAYLMGFQKL---DG 406

fx|XP3638|FusariumOxysporum NVLSVGIKSNT----------------DTATLSGTSMASPHVAGLAAYLMGFQQL---DG 406

fx|XP3639|FusariumOxysporum NVLSVGIKSNT----------------DTATLSGTSMASPHVAGLAAYLMGFQQL---DG 406

fg|XP0525|FusariumGraminearum SIKSTWIGSDS----------------ATETLSGTSMACPHVAGLSLYLRAKEGL---KT 354

fx|XP1712|FusariumOxysporum NVKSAWYTSNS----------------ATNTISGTSMACPHVAGLSLYLRAKEGL---TT 360

fg|XP3315|FusariumGraminearum GILSTWYTSNT----------------ATNSISGTSMACPHVAGLALYLQVLEGL---ST 376

fx|XP9167|FusariumOxysporum NILSTWIGSNS----------------ATNTISGTSMACPHVAGLALYLQVLEGL---ST 379

fg|XP8464|FusariumGraminearum DIVSASWLSDN----------------GTYVEDGTSMACPHVAGLVLYAQSVYGI---VG 410

fx|XP9240|FusariumOxysporum NVVSASWLSDN----------------GTYIESGTSMATPHVSGLVLYAQSVYGI---TG 405

fg|XP0806|FusariumGraminearum SVLSAWYTSNS----------------ATNTISGTSMATPHIAGLVLYGISVKGV---SG 377

fx|XP5679|FusariumOxysporum AVLSAWYTSNS----------------ATNSISGTSMATPHIAGLVLYGISVNGV---SG 380

fg|XP1405|FusariumGraminearum GILSSGWKSDT----------------HTFTEDGTSMAAPHVAGLVLKEVWLRVF---LC 378

fx|XP4666|FusariumOxysporum GITSAGHKSNT----------------YTFTEDGTSMAAPHVAGLVLYAMSVEEV---EG 379

ma|XP5016|MetarhiziumAcridum NVVTTTIGSDT----------------ATVTQSGTSLAAPHVAGLALYLITAENI---KT 378

mr|XP6566|MetarhiziumRobertsii NVMTISKDSDT----------------ATTVQSGTSLAAPHVAGLALYLIAAEKI---KT 378

cm|XP0589|CordycepsMilitaris NVVTISSA-QT----------------GPVRVDGTSFASPYVAGVALSLSALEKF---GS 370

cm|XP8384|CordycepsMilitaris DVLTISSK-EA----------------GAVRTSGTSLAAPYVAGIALYLCAVQNF---RT 405

: . . :

bb|XP7677|BeauveriaBassiana SEYAVASITPAELKDNIIS-IG--TKNSLSDIDS-ETPNVLAWNGAGCSDYKKIVQAGG- 470

cm|XP6358|CordycepsMilitaris SEYAVASITPAELKDNLIS-IG--TKNILSDIDN-DTPNVLAWNGAGCSDYKKIVEAGA- 470

pc|XP3125|PochoniaChlamydosporia SEYSVAPITPKQLKDTLIK-IS--TEGVLTDIPA-GTPNKLAWNGGGCSNYSKIVAAGG- 467

ma|XP0725|MetarhiziumAcridum SEFSVAPITPKQLKDTLIE-IS--TQGVLTDIPK-DTPNKLAWNGGGCSNYSKIVAAGG- 470

mr|XP6449|MetarhiziumRobertsii SEFSVASITPKQLKDTLIE-IS--TQGVLTDIPN-DTPNKLAWNGGGCSNYSKIVAAGG- 470

fg|XP0192|FusariumGraminearum SEYALASITPKKLKENLIS-VA--TENALSDIPS-DTPNLLAWNGGGCSDYKKIVEAGS- 468

fx|XP5893|FusariumOxysporum SEYALASITPKKLKENIIS-VA--TEDALSDIPS-DTPNLLAWNGGGCSDYKKIVEAGS- 467

pl|XP1616|PurpureocilliumLilacinum SEYSVAPITPKKLKETLIE-IS--TEGVLSDIPA-DTPNKLAWNGGGCNNFSKIVSAGG- 471

ti|XP5718|TolypocladiumInflatum SEYAVAPITPKKLKSSLIS-IA--TEGALSDLPE-DTPNKLAWNGGGCDNFSKIVEAGG- 466

tr|XP6166|TrichodermaReesei SEFAVAPITPKKLKESVIS-VA--TKNALSDLPDSDTPNLLAWNGGGCSNFSQIVEAGS- 471

ma|XP5931|MetarhiziumAcridum ------------------------------------------------------------ 354

mr|XP4522|MetarhiziumRobertsii ---------GPDLCAWMVQ-RA--TPQW-------------------------------- 386

ma|XP4373|MetarhiziumAcridum ---------VPKLCQYMKD-VA--VEGAVKEQN-LYTANLLVTNSVVGA----------- 411

mr|XP9378|MetarhiziumRobertsii ---------IPNMCQYLKD-VA--VKGAVKEQK-LYTANLVATNAVVDV----------- 417

bb|XP1899|BeauveriaBassiana ---------P-DICSTIQD-SA--TANAIVDQI-RGTKNLIAFNGNPRG----------- 397

cm|XP5458|CordycepsMilitaris ---------F-GLCQAIQQ-MA--TPNAIVDQV-WGTRNLLAFNGASQW----------- 437

bb|XP2589|BeauveriaBassiana ---------AAGMCEFLQN-IS--VKNAISGMH-YDTKNLLAQNDRAR------------ 400

cm|XP9564|CordycepsMilitaris ---------ADGLCEYLQS-IA--IKDAIWGVH-YGTKNLLAQNDMAQ------------ 392

pl|XP3276|PurpureocilliumLilacinum ---------AEPLCKRIQE-LA--TKDVIQNIP-KGTLNLLAFNGNPTG----------- 405

pl|XP1155|PurpureocilliumLilacinum ---------G-SACDRIQE-LA--TEGAISGIP-AGTVNLLAFNGNPSG----------- 389

pl|XP4677|PurpureocilliumLilacinum ---------AQALCERIRT-LS--TKNVLTGIP-SGTVNYLAFNGNPSG----------- 390

pl|XP9521|PurpureocilliumLilacinum ---------G-AICDRIKE-LA--TKDVITELLSPNTPNLLAFNGNPSG----------- 375

pl|XP0926|PurpureocilliumLilacinum ---------G-AVCDRIRA-LA--NKGILKNIQDAGSPNLLVFNGYPAAT---------- 392

ma|XP1723|MetarhiziumAcridum ---------PAKVMSRLRE-LADDTGARVQWTA-PNTTSLIATNGLAGKLDPAIANKVKK 380

mr|XP6087|MetarhiziumRobertsii ---------PSQVAARLKD-IAKQSGAQVQWNA-PHTTGLIASNGLDKG-GPSSLFPPKR 339

mr|XP3684|MetarhiziumRobertsii ---------AANIKKFLLE-KA--TKDRA-CVS-KSTPNLVANNGNAMQDKVKSDDKS-- 374

ma|XP0847|MetarhiziumAcridum ---------ATKIKQFLLQ-SA--TRDQA-CTY-HNTPNIVANNGNTVQKKHTKPRNC-- 372

mr|XP4016|MetarhiziumRobertsii ---------AAEIRKFLLQ-SA--TKDQA-CTS-RNTPNIVANNGNTAQKKHTKPRNC-- 372

bb|XP2820|BeauveriaBassiana ---------RRHEIVSMLDSTC--TKDKITGDL-RGSPNKLVNNNNKKQAK--------- 324

bb|XP0462|BeauveriaBassiana ---------RRHDIVSLLDSTC--TKDKIKGDL-RGSPNKLVNNNNKKQEK--------- 321

ma|XP3188|MetarhiziumAcridum ---------N-EVKFYLG-QTA--TKDMIKGDL-RGAPNLLVNNNNNEQESCERRSQPKN 342

bb|XP3476|BeauveriaBassiana ---------T-QVRWFLAEETA--TKDKIQGDL-GGAPNRLVNNNNSKQDSCKPK-EPKN 342

bb|XP0639|BeauveriaBassiana ---------T-QVRWFLVEETA--TKDKVQGNL-RGAPNRIANNNNSKQDSCALK-DPKN 343

ma|XP5324|MetarhiziumAcridum ---------PANVTARLKQ-LSGNTQAQVGNGK-SGTTPLIANNGNQKDKNE---FLTGD 468

mr|XP6435|MetarhiziumRobertsii ---------PAKVTALLKG-QAKETDATVEGGR-RDTTPLIANNGNQLDKNK---FLDEN 430

bb|XP8622|BeauveriaBassiana ---------PAQVARLITG-LADRSGDEVRRND-PQTTDRIATNTIG------------- 431

cm|XP2839|CordycepsMilitaris ---------PSQVEDLMKN-LAGQTGARVRGNE-QGTTNRIANNGSR------------- 425

pl|XP3880|PurpureocilliumLilacinum ---------ASEMSDMLKS-LASNSPARVKNIA-QGTSPLIVNNGNLS------------ 418

pc|XP9521|PochoniaChlamydosporia ---------IDDVNTMMQQ-LAQKTNAQVRNNV-PGTTSRIANNGF-------------- 422

ti|XP8977|TolypocladiumInflatum ---------PAQLDGLIKN-LASKTGATCSSNV-PGTTNLIANNGNL------------- 422

tr|XP7158|TrichodermaReesei ---------VDDVSNLIKN-LAAKTGAAVKQNI-AGTTSLIANNGNF------------- 387

fg|XP9382|FusariumGraminearum ---------PAQVASLIKS-LAAESGAKVRNNV-RGTTDGIANNGNQ*------------ 442

fx|XP3638|FusariumOxysporum ---------PAQVASLIKS-LAGQTGAKVQNNV-QGTTDSIANNGNQ------------- 442

fx|XP3639|FusariumOxysporum ---------PAQVASLIKS-LAGQTGAKVQNNV-QGTTDSIANNGNQ------------- 442

fg|XP0525|FusariumGraminearum ---------VKSVQDRIKQ-LA--TKNVIANAG-AGSPNLLAYNGGVPTRTT---FRKGD 398

fx|XP1712|FusariumOxysporum ---------PESVARRLKE-LA--TSGVVQDAG-SGSPNLLAYNGAPSS----------- 396

fg|XP3315|FusariumGraminearum ---------PAAVTNRIKA-LA--TTGRVTGTL-NGSPNLIAFNGAST*----------- 411

fx|XP9167|FusariumOxysporum ---------PASVTNRIKS-LA--TTGRITGTL-SGSPNSVAYNGNGA------------ 414

fg|XP8464|FusariumGraminearum ---------VKSTTNFIKK-YA--TNNKIVGSR-RGSPNRIANNNNLAQTK*-------- 448

fx|XP9240|FusariumOxysporum ---------VASTTKYIKK-YA--TTNKIVGSR-RGSPNRIANNNNFAQSR--------- 443

fg|XP0806|FusariumGraminearum ---------VSGVTNWLTS-TA--TSGKITGNL-RSSPNLIGNNGNSLQ*---------- 413

fx|XP5679|FusariumOxysporum ---------VSGVTNWLTS-TA--TSGQITGNL-RSSPNLIGNNGNTAQ----------- 416

fg|XP1405|FusariumGraminearum ---------VY*------------------------------------------------ 380

fx|XP4666|FusariumOxysporum ---------VADITAWLKE-LA--TPKKISGNL-RGAPNLIANNGNGVQ----------- 415

ma|XP5016|MetarhiziumAcridum ---------AAELRARILA-LA--TKNKVTNVP-ANTVNLLASNRAQ------------- 412

mr|XP6566|MetarhiziumRobertsii ---------PAELRARILA-LA--TKDKITNVP-ANTVNLLAFNGVQ------------- 412

cm|XP0589|CordycepsMilitaris ---------VQELVDRIKA-LG--TAGKVTGLP-PNTVNLVAFNGAPSA----------- 406

cm|XP8384|CordycepsMilitaris ---------AKDLTDRIKA-LG--TPGKASNLP-ANTVNLVAYNGAPALDAGTVVFEPGR 452
